# Bispecific nanobodies targeting Bet v 1 and ICAM-1 decrease respiratory epithelial penetration of Bet v 1

**DOI:** 10.3389/fimmu.2026.1864741

**Published:** 2026-08-17

**Authors:** Ines Zettl, Isabella Ellinger, Mohammed Zghaebi, Sarah Jance, Vanessa Izewski, Anja Drescher, Heimo Breiteneder, Manuel Röck, Martin Tollinger, Julia Eckl-Dorna, Sergei V. Tillib, Sabine Flicker

**Affiliations:** 1 Institute of Pathophysiology and Allergy Research, Center for Pathophysiology, Infectiology and Immunology, Medical University of Vienna, Vienna, Austria; 2 Vienna Airway Lab, Department of Otolaryngology, Head and Neck Surgery, Medical University of Vienna, Vienna, Austria; 3 Cytiva Europe GmbH, Freiburg, Germany; 4 Department of Organic Chemistry, University of Innsbruck, Innsbruck, Austria; 5 Institute of Gene Biology, Russian Academy of Sciences, Moscow, Russia

**Keywords:** birch pollen allergy, bispecific nanobody, ICAM-1, respiratory epithelium, topical treatment

## Abstract

**Background:**

The major birch pollen allergen Bet v 1 primarily affects the nose and eyes of allergic patients. After crossing the epithelial barrier, it causes IgE-mediated effector cell release, triggering inflammatory responses. We sought to develop bispecific nanobodies that bind Bet v 1 on mucosal surfaces to prevent allergen penetration and subsequent activation of effector cells. Intercellular adhesion molecule 1 (ICAM-1), highly upregulated on epithelial cells of allergic individuals, was chosen as an anchor.

**Methods:**

Genetically fused nanobodies targeting Bet v 1 and ICAM-1 were generated and characterized for specificity, affinity, Bet v 1 immobilization on the surface of the human bronchial epithelial cell line, 16HBE14o-, and their potential to inhibit Bet v 1 penetration through a cell monolayer.

**Results:**

Bispecific nanobodies recognized ICAM-1, Bet v 1 and related tree pollen allergens (Aln g 1, Cor a 1). While we observed a clear co-localization of nanobodies and Bet v 1 on the 16HBE14o-cell surface after 30 min, Bet v 1 was slightly internalized after 4h at 32 °C. Dissociation rate constants between bispecific nanobodies and antigens were 3–6 times faster at 32 °C compared to 25 °C. Bispecific nanobodies reduced Bet v 1 penetration and hence decreased basophil activation.

**Conclusion:**

We generated bivalent nanobodies specific for ICAM-1 and Bet v 1. These nanobodies diminished trans-epithelial transport of Bet v 1 by ICAM-1-mediated immobilization at the cell surface. Reduced Bet v 1 penetration resulted in decreased basophil activation, demonstrating the potential of local administration of nanobodies for the prevention of pollen allergy.

## Graphical Abstract

**Figure:**
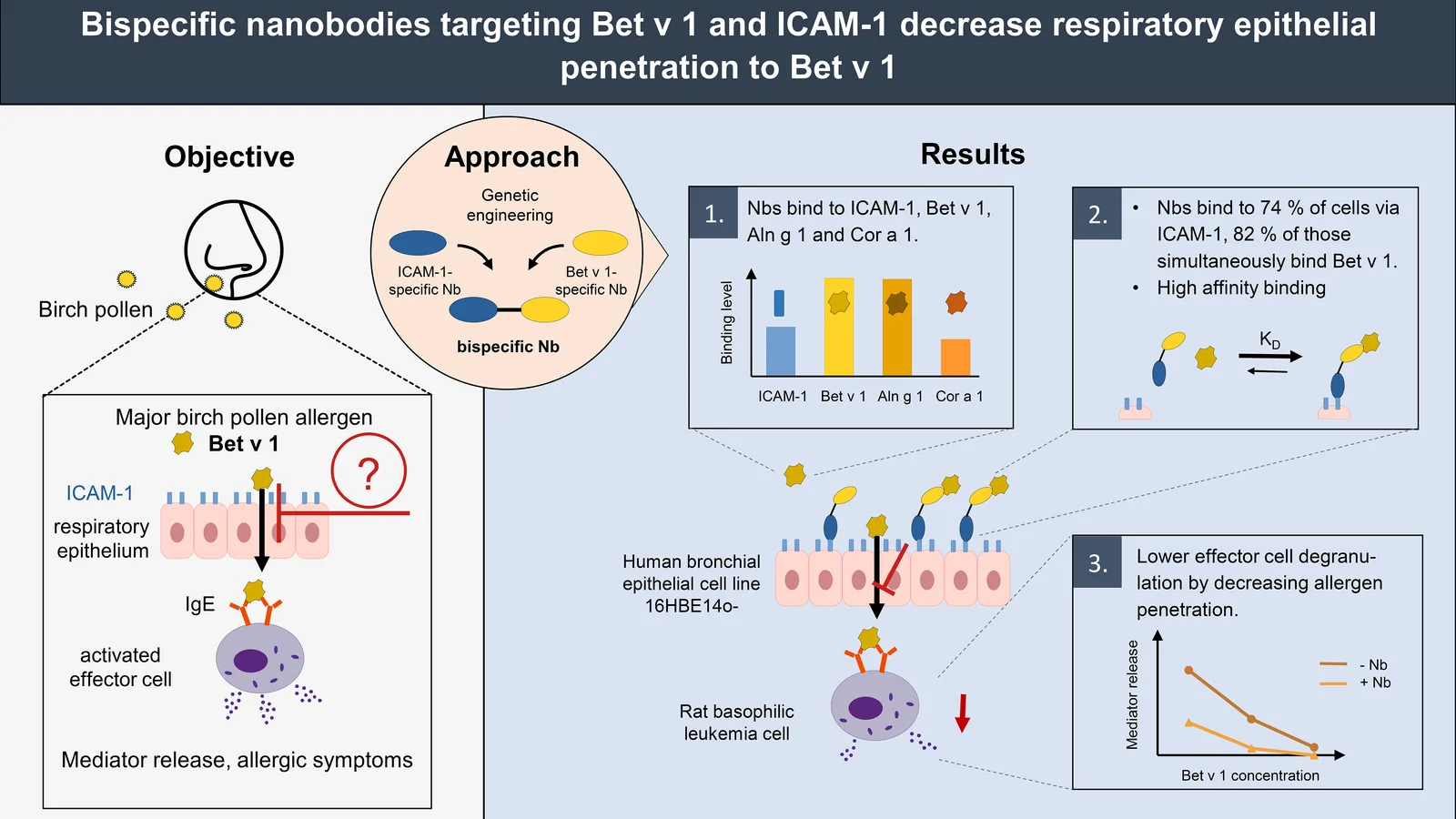

## Introduction

Birch pollen allergy is very prominent in the northern hemisphere (1–4). More than 100 million people suffer from birch pollen-induced allergic symptoms ranging from rhinoconjunctivitis to asthma during the spring months, which results in a huge socioeconomic burden for affected individuals (5–7). The main initiator of birch pollen-triggered pollinosis is Bet v 1, the sole major allergen (8). Together with its relatives present in tree pollen and pollen-associated plant foods, this aeroallergen is responsible for eliciting clinical manifestations in 95% of birch pollen-allergic patients (8–11)Airborne allergens like Bet v 1 primarily enter the body via the upper respiratory tract and the eyes. After crossing the epithelial barrier, they activate local effector cells such as basophils and mast cells by crosslinking receptor-bound IgE, resulting in the release of inflammatory molecules (12–14). The mechanisms behind the uptake of respiratory allergens are still under debate, including transcytosis which is mainly reported for allergic patients (15, 16), paracellular allergen penetration by loss of intercellular tight junctions (17–21) and uptake by dendritic cell extensions through tight junctions into the nasal cavity (22, 23).

Since the incidence and prevalence of respiratory allergy have rapidly increased in the last decades, the need for action is evident. Besides treatment and therapy to mitigate allergic disorders, great effort has been put into allergy prevention (24). Approaches to inhibit the trans-epithelial uptake of airborne allergens are a long-pursued goal to avoid allergic reactions and potential progression of respiratory allergies to chronic manifestations. Current strategies for allergen blocking rely on topically applied pharmacological and physical interventions to either strengthen the natural epithelial barrier or introduce a protective shield to support the barrier´s integrity (25, 26). In addition to treatment with intranasal corticosteroids and antihistamines to reduce local inflammation (25), various non-medical concepts have been developed (26). These measures range from nasal irrigation and wearing masks to recommendations to follow pollen forecasts. An individual wearable air purifier was recently evaluated for feasibility (27). However, these applications are either unspecific and/or are recommended as a supplement to pharmacological interventions. In contrast, we have previously proposed a biological shield made of allergen-specific antibodies as a specific and stand-alone option (28). By immobilizing monoclonal antibodies specific for the major timothy grass pollen allergens, Phl p 2 or Phl p 5, on the apical cell surface of respiratory epithelial cells, allergens were captured and the trans-epithelial allergen penetration and subsequent degranulation of basophils were reduced (28, 29). These allergen-specific antibodies were chemically linked to antibodies specific for the intercellular adhesion molecule-1 (ICAM-1) to properly anchor the conjugates to the cell surface. ICAM-1 is a well-known cell surface receptor that is upregulated in nasal and ocular epithelium cells, especially in allergic patients (30, 31). It was therefore considered a suitable target for this objective.

To refine this concept and to generate defined antibody formats comprising equal and reproducible functional efficiency, we decided to use single-domain antibodies (i.e., nanobodies, trademarked by Ablynx/Sanofi) as binding moieties for ICAM-1 and Bet v 1. Due to their modular and simple structure, nanobodies are well-suited as building blocks for larger and multi-specific constructs, and their production tends to be cheaper than that of monoclonal antibodies (32, 33). We have previously developed highly affine nanobodies against Bet v 1 and ICAM-1 (34, 35). Our generated Bet v 1-specific nanobody exhibit IgE blocking capacity (34). Furthermore, our selected ICAM-1-specific nanobody remained bound to the cell surface for several hours without being internalized to the cytoplasm (35). Here, we report on the construction and functional characterization of the first bispecific nanobody variants thereof. We investigated their binding behavior to both target proteins and two Bet v 1-homologous tree pollen allergens. In addition, we tested their ability to capture Bet v 1 on the cell surface and finally evaluated whether they could inhibit trans-epithelial allergen penetration and consequently the degranulation of effector cells.

## Materials and methods

### Antigens, cells and antibodies

Recombinant allergens from birch (Bet v 1), alder (Aln g 1), hazel (Cor a 1) and grass pollen (Phl p 5) were purchased from Biomay (Vienna, Austria). Recombinant Bet v 1 was also kindly provided by Heimo Breiteneder, Institute of Pathophysiology and Allergy Research (IPA), Medical University of Vienna (MUV). Recombinant Aln g 1 was a gift from Barbara Bohle, IPA, MUV. Additionally, recombinant Cor a 1 was produced and provided by Manuel Röck and Martin Tollinger, Department of Organic Chemistry, University of Innsbruck. Recombinant human ICAM-1 (Cat. No. ADP4), corresponding to the extracellular domains 1-5, was obtained from R&D Systems (Minneapolis, MN, USA). Unless otherwise specified, the chemicals used in this study were acquired from Merck Group (Darmstadt, Germany). The pMES4 vector was bought from Addgene (Watertown, MA, USA). *E. coli* strain WK6 for nanobody expression was from LGC Standards (ATCC-47078, Wesel, Germany). The human bronchial epithelial cell line 16HBE14o- was purchased from Merck Group (SCC150). RS-ALT8, a rat basophilic leukemia (RBL) cell line expressing the human high-affinity IgE receptor (FcϵRI) was kindly provided by Ryosuke Nakamura from Teikyo University (Kawasaki, Japan) (36). Information on antibodies used (ELISA, Western blot, flow cytometry, immunofluorescence staining) can be found in **Supplementary Table 1**. Allergen-specific rabbit sera were obtained by immunization of rabbits with purified recombinant allergens (Bet v 1, Phl p 5, ICAM-1) using complete and incomplete Freund adjuvant (CFA, IFA), respectively (Charles River, Kislegg, Germany). After informed consent was given, sera were obtained from patients suffering from birch pollen allergy according to case history, skin prick testing and positive a Bet v 1-specific IgE serology. Demographic, clinical and serological data of individuals were previously published (37). Serum samples were analyzed in an anonymized manner with permission from the Ethics Committee of the Medical University of Vienna (EK1758/2012, EK1760/2023).

### Cell culture

The human bronchial epithelial cell line 16HBE14o- was used as a model for the respiratory epithelium. The cells were cultured in minimum essential medium (MEM) (Cat. M2279, Sigma-Aldrich, St.Louis, MO, USA) with Earl’s salts and L-glutamine (Cat. 25030, Gibco, Life Technologies, Carlsbad, CA, USA) supplemented with 10% HyClone fetal bovine serum (FBS) (Cat. SH30071, Cytiva, Uppsala, Sweden) and 100 U/mL penicillin-streptomycin (Cat. 15140, Gibco) in an incubator set to 37 °C, 5% CO_2_ and 95% humidity according to the manufacturer’s instructions. Culture flasks were coated beforehand with 30 µg/mL bovine collagen type I (Cat. 354231, Corning, Corning NY, USA) and 10 µg/mL human fibronectin (Cat. 354008, Corning) in MEM (Cat. M2279, Sigma-Aldrich) supplemented with 0.01% bovine serum albumin (BSA) (Roth, Karlsruhe, Germany) for optimal growth conditions. Cells were split every 3–5 days. After washing with Dulbecco’s phosphate-buffered saline (DPBS, 1x) (Cat. 14190, Gibco), cells were detached with a solution of 0.05% trypsin and 0.02% ethylene-diamine-tetraacetic acid (EDTA) (Cat. T3924, Sigma-Aldrich) and taken up immediately in culture medium containing 10% FBS to quench the enzymatic activity of trypsin. After centrifuging (300 x g, 5 min), cells were resuspended in fresh medium and added to the culture flask, diluted 1/5 to 1/10 of their original density. Cells were used within six passages after thawing.

RBL cells (RS-ALT8) (36) transfected with FcϵRI were cultured in MEM (Cat. 31095, Gibco) supplemented with 10% heat-inactivated FBS (Cat. 16140, Gibco), 100 U/mL penicillin-streptomycin (Cat. 15140, Gibco), 2 mM L-glutamine (Cat. 25030, Gibco), 0.2 mg/mL Geneticin (Cat. CP12-2, Carl Roth, Karlsruhe, Deutschland), 0.2 mg/mL hygromycin B (Cat. 2039.2, Carl Roth) at 37 °C, 5% CO_2_ and 95% humidity. Cells were split every 3–5 days by detaching them with a cell scratcher (TPP, Trasadingen, Switzerland) and diluted 1/5 to 1/10 of their original density. Cells were used within six passages after thawing.

### Bispecific nanobody design, expression and purification

**Figure 1:**
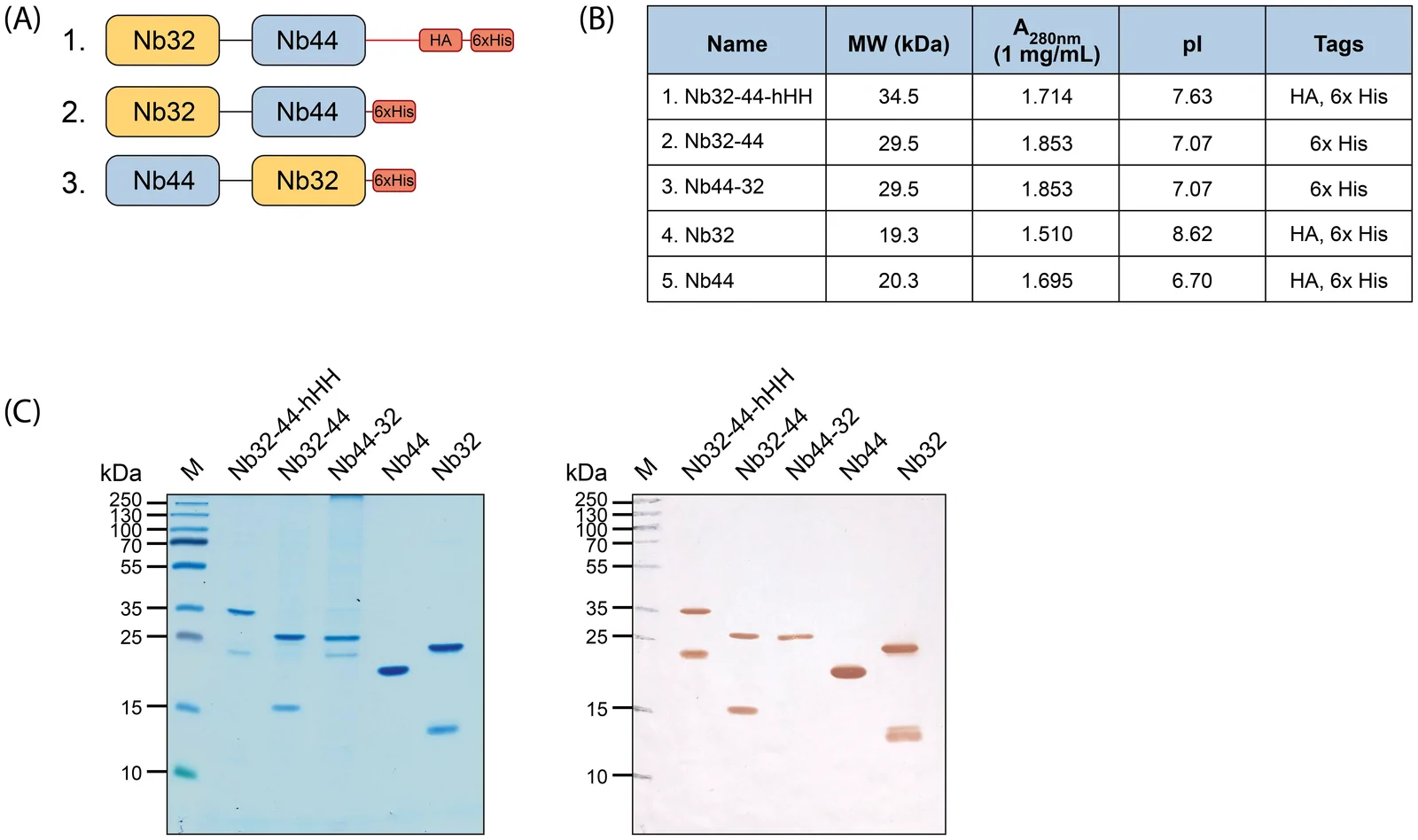
Biophysical characteristics of bispecific nanobodies. **(A)** Schematic representation of the three bispecific nanobody designs consisting of a Bet v 1-specific Nb (Nb32, yellow), an ICAM-1-specific nanobody (Nb44, blue) and attached tags (red). The red line in 1. represents the long camel hinge region. **(B)** Molecular weight (MW), specific absorption at 280 nm for a 1 mg/mL solution, isoelectric point (pI) and tags are listed for each bispecific construct and for Nb32 and Nb44. **(C)** Coomassie Blue-stained and blotted nanobodies (1 µg) under non-reducing conditions. Molecular weights in kDa are indicated on the left margins. Gels and blots were repeated three times.

We have previously generated nanobodies specific for Bet v 1 (Nb32) and ICAM-1 (Nb44) (34, 35). DNA sequences of Nb32 (https://www.ncbi.nlm.nih.gov/genbank, GenBank accession number MZ708607) and Nb44 (GenBank accession number ON950079) were linked head-to-tail with a standard (GGGGS)_3_ linker and equipped with a 6x His-tag. These constructs were designated Nb32–44 and Nb44-32, according to the terminal positions of the nanobodies (N-terminal first, C-terminal second) (**Figure 1A**). For the first construct (Nb32-44), another version was created, including the long camel IgG2 hinge region as a spacer between the C-terminus of the nanobody and the His-tag, and an additional HA-tag (designated Nb32-44-hHH) (**Figure 1A**). DNA sequences were standard codon-optimized for *E. coli* (38), synthesized and inserted (by ATG biosynthesis, Merzhausen, Germany) into the pMES4 vector. The full sequences were cross-checked by custom DNA sequencing (Eurofins Genomics, Ebersberg, Germany). Plasmids were transformed into electrocompetent WK6 cells and grown on LB-Agar plates with 100 µg/mL ampicillin (Cat. K029.1, Carl Roth) and 1% glucose overnight at 37 °C. Single colonies were inoculated in 30 mL 2x YT medium completed with 100 µg/mL ampicillin, 1% glucose, 2 mM MgCl_2_, 50 mM Na_2_HPO_4_, 50 mM KH_2_PO_4_ and 25 mM (NH_4_)_2_SO_4_ overnight at 30 °C. The next day, the preculture was centrifuged (2,300 x g, 10 min), resuspended in 6 mL fresh medium and added to 1 L complete 2x YT medium. After the optical density (OD) at 600 nm reached 0.6-0.7, nanobody expression was induced with 0.5 mM isopropyl-β-D-thiogalactopyranoside (Cat. CN08.2, Carl Roth) for 4 h at 37 °C. Afterwards, cells were pelleted (2,300 x g, 15 min, 4 °C) and frozen overnight. Pellets were thawed, resuspended in 12 mL TES (200 mM Tris/HCl, 5 mM EDTA, 500 mM saccharose) supplemented with 100 µg/mL phenylmethanesulfonyl fluoride (Cat. 78830, Sigmna-Aldrich) and incubated shaking for at least 2 h on ice. The cell suspension was processed with a LV1 low-volume microfluidizer homogenizer (Microfluidics, Westwood, MA, USA) or a MM 200 oscillating mill (Retsch, Haan, Germany) for disrupting the cell membranes and afterwards 24 mL of 5 mM MgSO_4_ was added for another 30 min on ice. Cells were centrifuged (24,000 x g, 30 min, 4 °C) and the supernatant was purified with HIS-Select^®^ Nickel Affinity Gel (Sigma-Aldrich). Nanobodies were eluted with elution buffer (50 mM NaH_2_PO_4_, 300 mM NaCl containing 250 mM imidazole), spin filtrated using Amicon Ultra-15 centrifugal filter tubes (3,000 Da cut-off, Merck Millipore, Billerica, MA, USA) and buffer-exchanged into 1x Phosphate-buffered saline (PBS). Protein concentration was determined by absorption at 280 nm (DeNovix DS-11 FX+ spectrophotometer, Wilmington, DE, USA). Theoretical isoelectric points, molecular masses and extinction coefficients were calculated based on the amino acid compositions using ProtParam by Expasy (https://web.expasy.org/protparam/).

### SDS-PAGE and Western blot

Sodium dodecyl sulfate polyacrylamide gel electrophoresis (SDS-PAGE) and Western blot were performed as previously described (34, 35). Briefly, purified nanobodies were separated on a 14% polyacrylamide gel under reducing and non-reducing conditions. Protein bands were stained with InstantBlue Coomassie Protein Stain (Abcam, Cambridge, UK). The PageRuler™ Plus Prestained Protein Ladder, 10 to 250 kDa from Thermo Fisher Scientific (Cat. 26619) was used for size comparison. For Western blots, separated nanobodies were transferred to a nitrocellulose membrane (0.2 µm. Amersham Protran, GE Healthcare, Chicago, IL, USA) and detected with horseradish peroxidase (HRP)-conjugated goat anti-His-tag antibodies (PA1-23024, Invitrogen, Carlsbad, CA, USA) diluted 1:5,000 in buffer A (40 mM Na_2_HPO_4_,0.6 mM NaH_2_PO_4_, 0.5% BSA, 0.5% Tween 20, and 0.05% NaN3) and HRP substrate 3´,3´-diaminobenzidine (DAB) (Cat. CN75.1, Carl Roth).

### Reactivity of bispecific nanobodies (ELISA)

Antigens (ICAM-1, Bet v 1, Aln g 1, Cor a 1) were coated onto ELISA plates (MaxiSorp, Nunc, Roskilde, Denmark) diluted in 0.1 M bicarbonate buffer (2 µg/mL) overnight at 4 °C. After washing with PBS containing 0.05% Tween20 (PBST), plates were blocked with 1% BSA in PBST and afterwards incubated with 1 µg/mL bispecific nanobodies diluted in 0.1% BSA in PBST overnight at 4 °C. ICAM-1-specific nanobody Nb44 (1 µg/mL) and Bet v 1-specific nanobody Nb32 (1 µg/mL) diluted in 0.1% BSA in PBST, served as positive controls. To assess target specificity, two-fold serial dilutions of the bispecific nanobodies (ranging from 0.063 µg/mL to 2 µg/mL) were evaluated for binding to Bet v 1 (2 µg/mL), with Phl p 5 (2 µg/mL) serving as a control for non-specific binding. Bound nanobodies were detected with HRP-labeled mouse anti-His-tag antibodies (Cat. MA1-21315, Invitrogen) diluted 1:4,000 in 0.1% BSA in PBST. After repeated washing, HRP substrate 2,2’-azino-bis(3-ethylbenzothiazoline-6-sulfonic acid) (ABTS) (Cat. A1888, Sigma-Aldrich) was added to achieve the color reaction. ODs at 405 nm (reference wavelength: 495 nm) were measured with a TECAN Infinite F50 microplate reader (Männedorf, Switzerland).

To investigate simultaneous binding to both targets (ICAM-1 and allergen), plates were either coated with ICAM-1 or allergens (2 µg/mL), blocked and incubated with bispecific nanobodies (1 µg/mL) as described above. Then, 1 µg/mL allergens or ICAM-1 (diluted in 0.1% BSA/PBST) were added overnight at 4 °C. For control purposes, buffer (0.1% BSA/PBST) was used instead of either bispecific nanobodies or the second antigen. The secondary antigens were traced with specific rabbit sera (Bet v 1-specific, 1:5,000 or ICAM-1-specific, 1:10,000 diluted in 0.1% BSA in PBST) for 3 h (room temperature) and then detected with HRP-labeled donkey anti-rabbit antibodies (Cat. SAB 3700934, Sigma-Aldrich) diluted 1:2,000 in 0.1% BSA in PBST and ABTS. ODs at 405 nm (reference wavelength: 495 nm) were measured with a TECAN Infinite F50 microplate reader.

### Flow cytometry

Flow cytometry for detecting bispecific nanobodies on 16HBE14o- cell surfaces was performed as previously described (35). Cells were gently detached using a brief incubation with Trypsin-EDTA (3–4 min, 37 °C). The reaction was immediately quenched with FBS-containing medium, and all subsequent processing steps were strictly performed at 4 °C to preserve cell surface receptor integrity and prevent the proteolytic degradation of ICAM-1. Cells (2 x 10^5^ cells per well) were then seeded in 96 well V-bottom plates (Cat. 651901, Greiner, Kremsmünster, Austria) were blocked with 10% mouse serum (Cat. M5905, Sigma-Aldrich) stained for live/dead distinction (Fixable viability dye eFluor 780, Thermofisher Scientific) and incubated with bispecific nanobodies (30 µg/mL), Nb44 (20 µg/mL) or Nb32 (20 µg/mL) for 20 min at 4 °C. Bound nanobodies were detected with PE-conjugated anti-His-tag antibody (Cat. 362603, Biolegend, Amsterdam, The Netherlands). In a second set of experiments, cells were additionally incubated with Alexa Fluor (AF) 647-labeled Bet v 1 after exposure to nanobodies. Bet v 1 labeling was carried out with an AF 647 Conjugation Kit (Fast)-Lightning-Link (Cat. Ab269823, Abcam) following the manufacturer’s protocol. Samples were analyzed on a BD LSRFortessa (BD Biosciences, Franklin Lakes, N J, USA), acquiring 20,000 cells per sample in triplicates, and were evaluated with FlowJo Software (version 10, FlowJoLCC, Ashland, USA). To optimize acquisition settings, compensation beads (UltraComp eBeads, Invitrogen) were used. The gating strategy was single cells, live cells, PE-positive cells, and AF 647-positive cells (**Supplementary Figure 1**).

### Immunofluorescence staining and fluorescence microscopy

16HBE14o- cells (2 x 10^4^ per well) were seeded on ibiTreat tissue culture-treated 8 well µ-slides (Cat. 80826, Ibidi GmbH, Munich, Germany) overnight at 37 °C. After washing three times with DPBS, cells were incubated with nanobodies (50 µg/mL in 1%BSA/MEM) for 30 min, 4 h or 24 h at 32 °C. To simulate the actual temperature of the nasal cavity that ranges from 30 °C in the nasal valve area to 34 °C in the middle turbinate (39), we examined nanobodies´ behavior at 32 °C. In some wells, lysine-fixable, fluorescein-conjugated dextran (40 kDa, 10 mg/mL, Cat.D1845, Thermofisher) was simultaneously added to the cells to visualize the endolysosomal pathway. In a second set of experiments, cells were first incubated with nanobodies (50 µg/mL) for 30 min, washed with DPBS and subsequently exposed to AF 488-labeled Bet v 1 (12.5 µg/mL in 1% BSA/MEM) for 30 min or 4 h at 32 °C. Bet v 1 labeling was performed with an AF 488 microscale protein labeling kit (Cat. A30006, Thermofisher Scientific) according to the company’s manual. Cells were then washed with DPBS and fixed with 4% formaldehyde solution (Cat. FB002, Invitrogen). After washing and quenching residual fixing solution with 50 mM NH_4_Cl in PBS, cells were blocked with 5% goat serum (Cat. 005-000-121, Jackson ImmunoResearch Laboratories, West Grove, PA, USA) in PBS and 0.05% saponin to allow permeabilization of detection antibodies. Nanobodies were detected with mouse anti-HA tag antibodies (diluted 1:500, Cat. 26183, Invitrogen) and visualized with AF 568-conjugated goat anti-mouse antibodies (diluted 1:1000, Cat. A-11004, Thermofisher). Cell nuclei were stained with DRAQ5 fluorescent-probe solution (25 µM, Cat. 62254, Thermofisher). In between incubation steps, cells were washed three times with DPBS. Staining steps were executed at room temperature. Processed samples were stored at 4 °C until imaging. Confocal microscopy imaging was performed with a UltraVIEW ERS Confocal Imager (Perkin Elmer, Waltham, MA, USA) connected to a Zeiss Axiovert 200 microscope fitted with a 63x/1.4 oil objective lens (Plan-Apochromat, Zeiss, Oberkochen, Germany). Fluorophores were excited using a 488/561/640 multiline argon/krypton laser. Pictures were digitized and processed by Volocity software (Version 5.5., Perkin Elmer). Representative images of the experimental conditions were further edited with Adobe Photoshop (Version 12.0.4) with identical conditions for all images.

### Affinity measurements using surface plasmon resonance

Affinity measurements were performed on a Biacore 1K (Cytiva, Uppsala, Sweden) at 25 °C and 32 °C at a flow rate of 30 µL/min. Bet v 1 diluted in 10 mM acetate buffer pH 5.0 was immobilized on the surface of a Sensor Chip CM5 (Cytiva) using amine coupling chemistry according to the company’s instructions. Despite the presence of multiple potential attachment sites, standard amine coupling was highly reproducible (34, 37) indicating that enough target epitopes remained sterically accessible and the immobilization did not negatively impact the binding kinetics. The reference cell was only activated and deactivated without immobilizing any antigen. Binding kinetics to Bet v 1 were determined by the single-cycle kinetics program. Bispecific nanobodies were injected sequentially in three-fold dilutions (Nb32-44-hHH: 3.6 nM to 870 nM, Nb32-44: 1.5 nM to 373 nM, Nb44-32: 3.9 nM to 948 nM) for two min followed by a short injection pause of one minute in between each concentration. Subsequently, HBS-P+ buffer (Cytiva, 10 mM HEPES, 150 mM NaCl and 0.05% Surfactant P20) was injected for 30 min to record the dissociation phase. Nb32 and Nb44 (0.7 nM to 174 nM, three-fold dilutions) were injected in the same way and used as positive and negative control, respectively. HBS-P+ injections served as blank cycles. All samples were injected as technical duplicates. Flow cells were regenerated with 20 mM NaOH for 30 seconds. Recorded data were analyzed with the Biacore Insight Evaluation Software (Version 5.0.18.22102) by subtracting the mean of the blank cycles and fitting the dissociation phase to a 1:1 binding model to calculate the dissociation rate constant (k_d_).

To investigate the interaction between Bet v 1-bound bispecific nanobodies and ICAM-1, multi-cycle (Nb32-44-hHH, Nb32-44) or single cycle (Nb44-32, for reasons of saving nanobody reagent) kinetic assays with a capture element were performed. Nanobodies (Nb32-44-hHH: 250 nM, Nb32-44: 316 nM, both diluted in HBS-P+) were captured on the Bet v 1 surface for five min to reach a level of approximately 20 response units (RU). ICAM-1, diluted in HBS-P+ two-fold ranging from 5 nM to 80 nM, was injected for two min, each followed by a dissociation phase of ten min. HBS-P+ instead of ICAM-1 was injected for blank cycles. Nb44-32 (186 nM, diluted in HBS-P+) was captured on the Bet v 1 surface for two min to reach a level of approximately 20 response units (RU). Subsequently, ICAM-1 (5 nM to 80 nM) in two-fold dilutions in HBS-P+ was injected for two min for each concentration followed by HBS-P+ buffer for ten min to record the dissociation phase. Flow cells were regenerated using 20 mM NaOH injected for 30 seconds. Recorded data were analyzed with the Biacore Insight Evaluation Software (Version 5.0.18.22102) by subtracting the mean of the blank cycles and fitting the dissociation phase to a 1:1 binding model to calculate k_d_.

### Trans-epithelial allergen penetration assays

Trans-epithelial penetration assays were performed with the Corning Costar Transwell system using 12 mm inserts with 0.4 µm pore polyester membrane (Cat. 3460, Corning, NY, USA), allowing a volume of 500 µL in the upper (apical) chamber and 1500 µL in the lower (basolateral) chamber. 16HBE14o- cells (2–4 x 10^5^ cells per insert) were seeded on the membrane of apical chambers in culture medium (MEM/L-Glu/FBS/PenStrep) and basolateral wells were filled with same culture medium. Confluency and the formation of tight junctions were checked by measuring trans-epithelial electrical resistance (TEER) with an EVOM^2^ volt-ohm meter (World precision instruments, Inc. Sarasota, FL, USA). Baseline resistance of empty apical wells (no cells) were between 140 Ωcm^2^ and 150 Ωcm^2^ and were subtracted from measured values (TEER_sample_-TEER_blank_) x surface area (cm^2^). Cells were considered confluent with a TEER of over 700 Ωcm^2^ which was usually reached 3 days after incubation at 37 °C. After reaching confluency, cells were washed twice with DPBS, and provided with different media as follows. Derived from healthy tissue, 16HBE14o- cells do not represent the pathologically altered epithelium characteristic of allergic patients. Due to the lack of disease-specific up-regulation of the caveolar transport machinery, active transcellular transport of Bet v 1 cannot be investigated with this cell model. To mimic the impaired epithelial barrier in allergic patients and analyze the paracellular penetration, some apical chambers were filled with MEM supplemented with the calcium chelator ethylene glycol tetraacetic acid (EGTA) (5 mM), a known disruptor of tight junctions (40). 16HBE14o- cells representing intact epithelial barriers and apical chambers without cells were maintained in MEM/10% FBS and subjected to the same washing steps as the EGTA-treated samples to ensure closely matched experimental conditions. In preliminary tests, we noted that FBS concentration above 2% added to MEM inhibited the initial permeation activity of 5 mM EGTA. Therefore, we omitted FBS to promote the opening of epithelial tight junctions through extracellular Ca^2+^ depletion. However, culturing 16HBE14o- cells in MEM without FBS for the entire experimental period (=27 h) lowered the TEER even in the absence of 5 mM EGTA. Based on our own and reported observations (40, 41), we selected MEM without FBS during the initial EGTA incubation and MEM/10% FBS + 5 mM EGTA during nanobody and allergen incubation to examine the paracellular penetration of Bet v 1. After 1 h of EGTA treatment, when TEER was below 200 Ωcm^2^, cells were washed with DPBS and incubated with 1.) 20 µg/mL (500-fold excess) or 40 µg/mL (1000-fold excess) Nb32-44-hHH in MEM/10% FBS supplemented with 5 mM EGTA (+Nb32-44-hHH), or 2.) MEM/10% FBS supplemented with 5 mM EGTA only (-Nb32-44-hHH). Untreated cells and chambers with no cells were incubated in MEM/10% FBS. After three h, cells of all conditions (untreated, +Nb32-33hHH, - Nb32-44-hHH, no cells) were washed with DPBS and then exposed to 20 ng/mL Bet v 1 in MEM/10% FBS supplemented with (+Nb32-44, -Nb32-44) or without (untreated, no cells) 5 mM EGTA for 24 h. To simulate the actual temperature of the nasal cavity, cells were exposed to 32 °C during the addition of nanobodies and allergens (=27 h). Furthermore, basolateral chambers were provided with MEM containing 2% FBS during addition of nanobodies and allergens because we identified during assay set-up that FBS concentrations above 2% added to MEM affected basophil activation read out. We followed the TEER of all conditions throughout the entire experiments. After the incubation time, supernatants from both apical and basolateral chambers were harvested and stored at -20 °C until further investigation.

### Degranulation assays with rat basophilic leukemia cells

Human FcϵRI-transfected RBL cells (1.5–3 x 10^5^ cells per well) were seeded in 96-well, flat-bottomed cell culture plates (Cat. 3595, Costar, Corning) and incubated with IgE-containing sera from birch pollen allergic patients diluted 1:10 in culture medium, or for control purposes with medium only, overnight at 37 °C. Cells were washed three times with Tyrode’s buffer (Tyrode’s Salts, Cat. T2145, Sigma-Aldrich) supplemented with 24 mM NaHCO_3_ and 0.1% BSA and then exposed to basolateral media from transwell experiments diluted in serial three-fold dilutions (1:3 – 1:135) in buffer A (50% D_2_O (Cat. 15188, Sigma-Aldrich) in Tyrode’s buffer and 0.1% BSA) for one h at 37 °C. To calculate the background level of each individual basolateral medium used in our assays, RBL cells not exposed to sera were incubated with basolateral medium of each chamber. These yielded values were subtracted from the corresponding release elicited by the basolateral medium with IgE-sensitized cells. Furthermore, RBL cells incubated with patients’ sera were also exposed to two distinct concentration of Bet v 1 (4.5 pM, 1.5 pM) to verify their reactivity. Non-sensitized cells were lysed with 1% Triton X-100 (Cat. X-100, Sigma-Aldrich) to induce 100% degranulation. After the incubation time, cells were spun down, supernatants (50 µL) were transferred to a transparent, flat-bottomed 96-well plate (Cat. 655101, Greiner) and mixed with an equal volume of 0.16 mM 4-methylumbelliferyl N-acetyl-β-D-galactosaminide (4-MUG) (Cat. M2133, Sigma-Aldrich) in 0.1 M citric acid buffer (pH 4.2). The reaction was stopped after one h at room temperature in the dark with stop buffer (0.2 M glycine, 0.2 M NaCl, pH 10.7). The amount of released β-hexosaminidase as determined by the reaction with 4-MUG was measured with fluorescence (excitation: 360 nm, emission: 465 nm) with a TECAN infinite M200pro plate reader. Absolute values were converted to percentages in relation to lysed cells.

## Results

### Nanobody design and expression

DNA sequences of a Bet v 1-specific nanobody (Nb32) and an ICAM-1-specific nanobody (Nb44) were linked head-to-tail via a standard (GGGGS)_3_-linker and equipped with a C-terminal 6x His-tag (Nb32–44 and Nb44-32) (**Figure 1A**). One bispecific nanobody-construct had an additional HA-tag and the long camel hinge region as a spacer before the tags (Nb32-44-hHH) (**Figure 1A**). The calculated molecular masses and theoretical isoelectrical points (pI) based on the amino acid sequences are 34.5 kDa and 7.63 (Nb32-44-hHH), and 29.5 kDa and 7.07 (Nb32-44, Nb44-32), respectively. For comparison, we included the calculated molecular masses and pIs of Nb32 (19.3 kDa, pI: 8.62) (34) and Nb44 20.3 kDa, pI: 6.70) (35) (**Figure 1B**). Nb32-44-hHH continuously yielded higher protein expressions compared to the other two designs (approximately 300 µg/L for Nb32-44-hHH vs 100 µg/L for Nb32-44/Nb44-32). Analysis of purified nanobodies by reducing and non-reducing SDS-PAGE and Western Blot revealed bands at the expected size of approximately 35 kDa for Nb32-44-hHH, while Nb32–44 and Nb44–32 migrated around 25 kDa (**Figure 1C**, **Supplementary Figures 2A–C**). We observed additional lower molecular weight bands for Nb32-44-hHH (approx. 20 kDa) and Nb32-44 (approx.15 kDa). The ICAM-1 specific Nb44 appeared as a single band at approximately 20 kDa, as shown earlier (35). Two bands were visible for the Bet v 1-specific Nb32 (approx. 23 kDa and 13 kDa), consistent with previous results (34) (**Figure 1C**). The additional protein band was also recognized via its His-tag, showing moderate efficiency in full-length Nb32 production.

### Bispecific nanobodies bind to their specific antigens and Bet v 1 homologs

**Figure 2:**
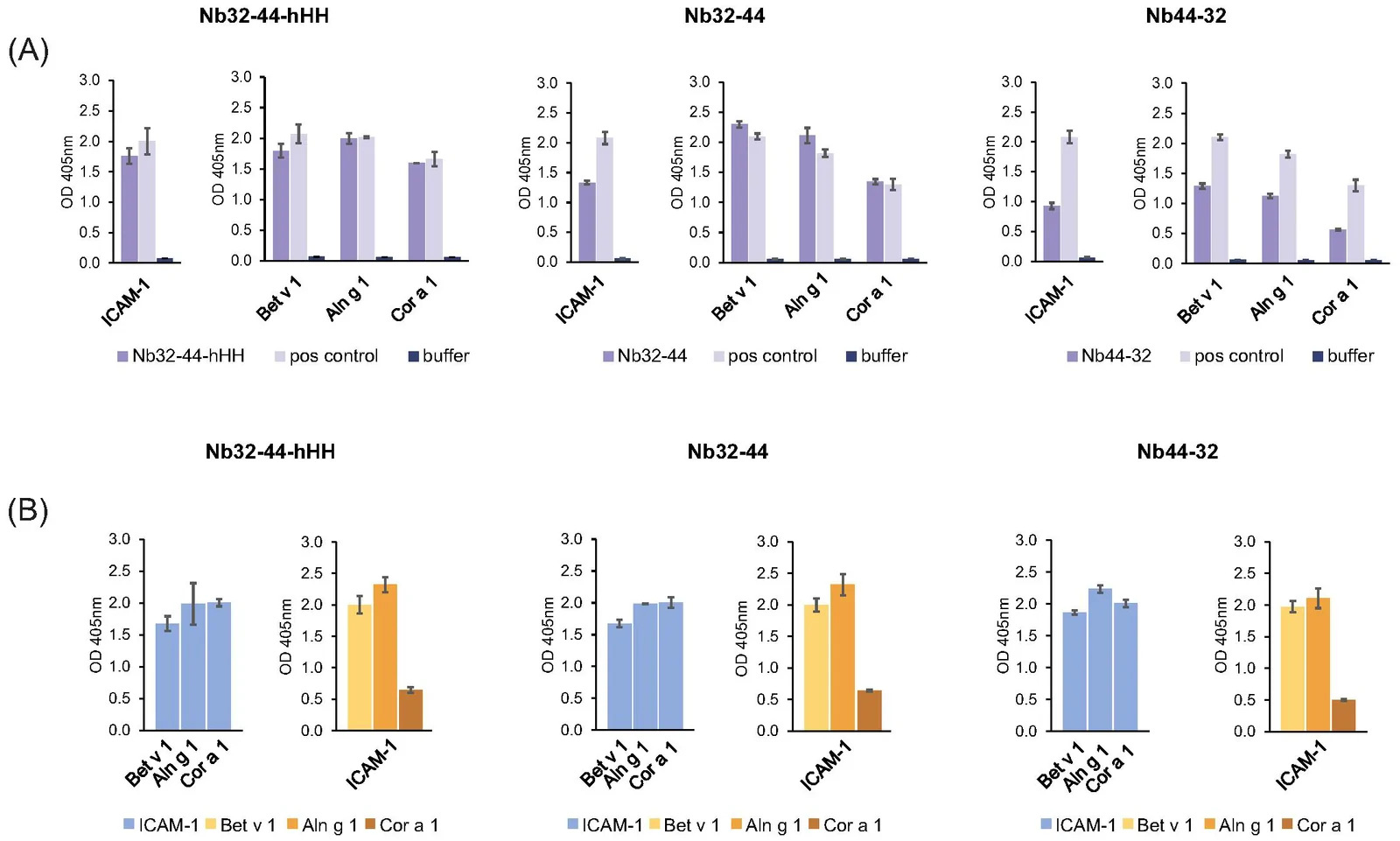
Binding capacity of bispecific nanobodies Nb32-44-hHH, Nb32–44 and Nb44–32 as determined by ELISA. **(A)** Reactivity of individual bispecific nanobodies with immobilized antigens indicated on the x-axes. Nb44 (for ICAM-1) and Nb32 (for allergens) served as positive controls (pos con). **(B)** Reactivity of ICAM-1 (orange) with bispecific nanobodies that were pre-bound to coated allergens and reactivity of allergens (blue) with bispecific nanobodies that were pre-bound to coated ICAM-1. OD-values correspond to the amount of bound nanobodies **(A)** or antigens **(B)** and are depicted as means of technical triplicates ± standard deviation (SD). Shown graphs are representatives of three biological replicates.

To verify that the nanobodies retained their original binding capabilities in the bispecific constructs, we performed ELISA with recombinant ICAM-1, Bet v 1 and Bet v 1 homologs Aln g 1 and Cor a 1. All three nanobody constructs bound to ICAM-1, Bet v 1, Aln g 1, and Cor a 1 (**Figure 2A**). However, it turned out that Nb44–32 displayed lower recognition across all antigens. Whether this finding is related to the weak expression quality of Nb44–32 or a suboptimal arrangement of the nanobody order of this construct needs further addressing. While Nb32-44-hHH bound equally well to all recognized antigens, Nb32–44 exhibited a stronger signal to the allergens than ICAM-1. Nb32 (Bet v 1, Aln g 1, Cor a 1) and Nb44 (ICAM-1) confirmed antigen immobilization to the plate (**Figure 2A**, pos control). Incubation of the antigens with detection antibodies only gave no signal (**Figure 2A**, buffer). To investigate whether bispecific nanobodies interact with both target antigens at the same time, we coated the plates either with allergens or ICAM-1 (**Figure 2B**). We confirmed that bispecific nanobodies immobilized to allergens were able to interact simultaneously with ICAM-1 (**Figure 2B**, blues graphs). When bound to ICAM-1, bispecific nanobodies recognize Bet v 1 (yellow graphs), Aln g 1 (orange graphs) and Cor a 1 (brown graphs). However, the response of all three ICAM-1 immobilized bispecific nanobodies to Cor a 1 was diminished. When bispecific nanobodies or the second antigens were omitted, no signal could be detected (**Supplementary Figure 3**). To confirm the absence of non-specific binding, we performed serial dilutions of all three bispecific nanobodies and evaluated their interaction with the unrelated major grass pollen allergen, Phl p 5. No binding was observed, demonstrating the high specificity of our constructs (**Supplementary Figure 4A**).

### Bispecific nanobodies capture Bet v 1 on the surface of bronchial epithelial cells

**Figure 3:**
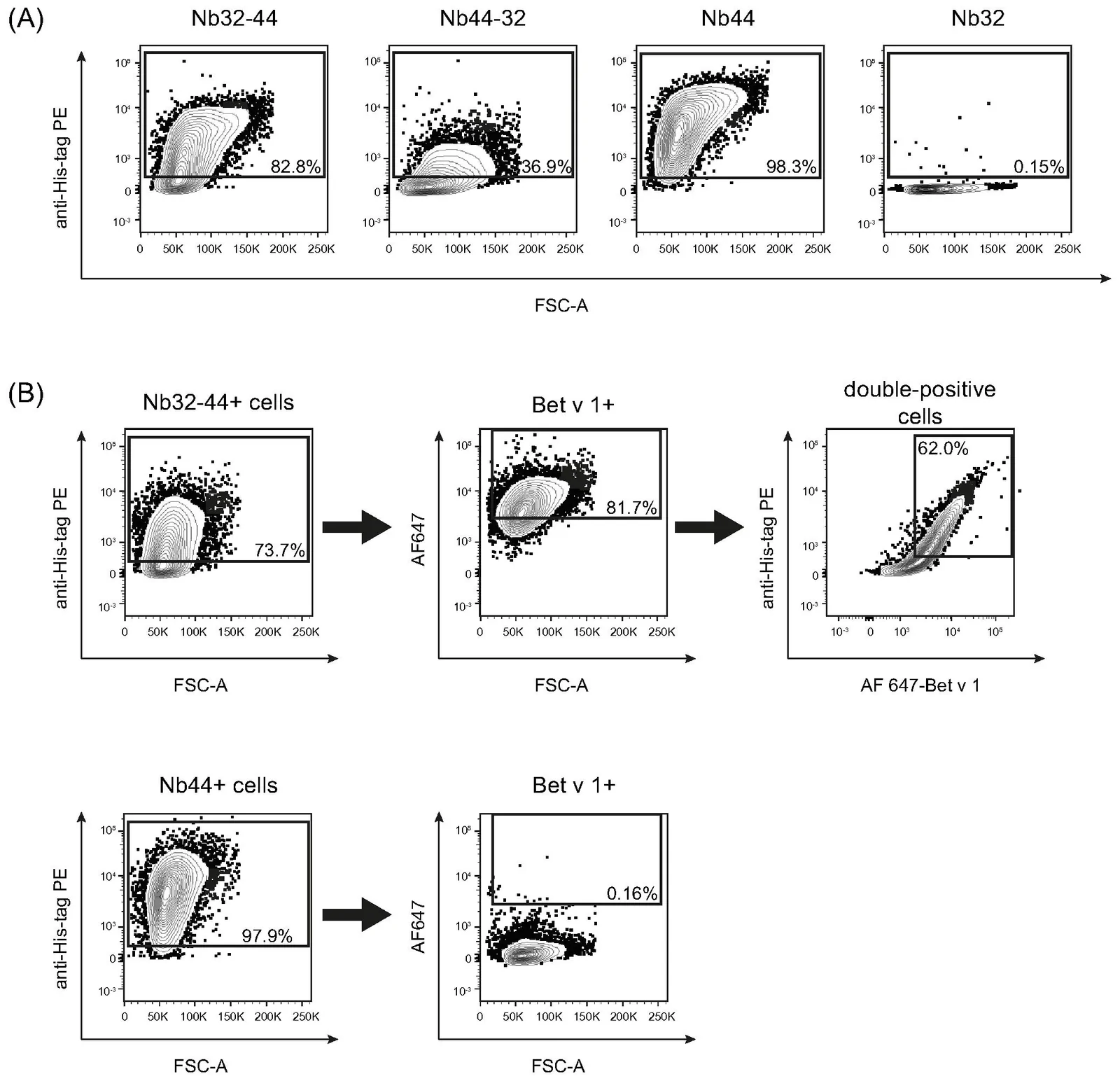
Flow cytometric analysis of bispecific nanobodies simultaneously binding to Bet v 1 and ICAM-1 on cell surfaces. **(A)** 16HBE14o- cells expressing ICAM-1 were incubated with bispecific nanobodies (Nb32-44, Nb44-32) or control nanobodies (ICAM-1-specific Nb44, Bet v 1-specific Nb32) at 4 °C. ICAM-1 bound nanobodies were detected with PE-labeled anti-His-tag antibodies. Representative plots of two (Nb32-44) and one (Nb44-32) independent experiments performed in quadruplicates show forward scattering (FSC-A, x-axis) versus the respective fluorophore (y-axis). **(B)** Cells were successively incubated with Nb32–44 and AF647-labeled Bet v 1 at 4 °C, and nanobodies were detected as described above. Representative plots of four independent experiments performed in quadruplicates show forward scattering (FSC-A, x-axes) versus the respective fluorophore (y-axis). Cells were gated on **(A)** single, alive and PE- (nanobody-) positive cells or **(B)** single, alive, PE-positive and AF647- (Bet v 1-) positive cells.

**Figure 4:**
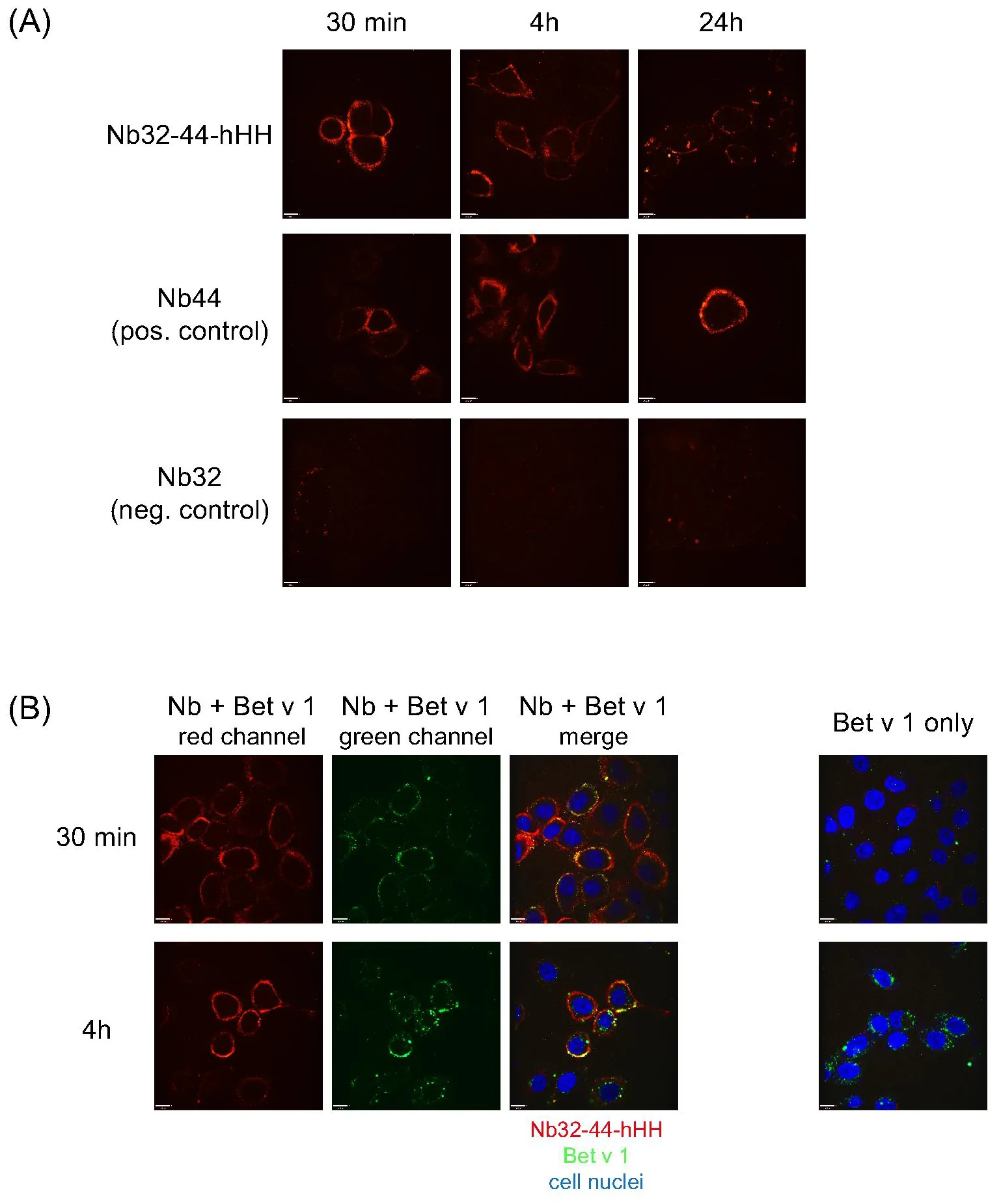
Confocal immunofluorescence microscopy of 16HBE14o- cells showing localization of Nb32-44-hHH, Nb32 and Nb44 (red) and Bet v 1 (green). **(A)** Cells were incubated with nanobodies for the indicated duration at 32 °C, fixed and stained with mouse anti-HA-tag antibodies and AF 568-labeled anti-mouse antibodies. **(B)** Cells were incubated with Nb32-44-hHH for 30 min and subsequently with AF 488-labeled Bet v 1 for 30min or 4h at 32 °C. After fixing, bound nanobodies were detected with AF 568-labeled anti-mouse antibodies. Cell nuclei were stained with DRAQ5. Yellow coloring indicates co-localization of nanobody (red) and Bet v 1 (green). Images shown are representatives of at least three independent experiments performed in duplicates (except A, 24h: one experiment). The white scale bar in the bottom left corners corresponds to 10µm.

To test whether bispecific nanobodies can bind to ICAM-1 expressed by the human bronchial epithelial cell line 16HBE14o- and furthermore can immobilize Bet v 1 on the cell surface, we performed flow cytometry as well as immunofluorescence staining followed by confocal microscopy. Flow cytometric analyses performed at 4 °C verified that the bispecific nanobodies recognized ICAM-1 on the cell surface. In accordance with our previous ELISA results, we found that Nb32–44 bound to a larger proportion of cells (78.6 ± 4.2%) than Nb44-32 (36.2 ± 0.8%) (**Figure 3A**). Therefore, we continued our investigation with Nb32–44 as it showed greater potential. As expected, Nb44 (positive control) bound 96.6 ± 4.3% of cells, whereas Nb32 (negative control) caused no signal (0.6 ± 0.4%) (**Figure 3A**). Next, we examined whether Bet v 1 could be captured by Nb32–44 already immobilized via ICAM-1 on16HBE14o- cells. Out of 82.7 ± 7.4% PE-positive cells (i.e., nanobody-bound), 74.0 ± 7.8% cells also emitted Bet v 1 signals (55.2 ± 6.7% double positive cells). When incubated with the monospecific ICAM-1 binder Nb44, Bet v 1 could not be detected on the cell surfaces (**Figure 3B**). To investigate possible internalization of the Bet v 1:nanobody complexes, we performed immunofluorescence staining and confocal microscopy at 32 °C. A temperature of 32 °C was chosen to reflect the actual temperature of the nasal cavity. Since we observed a high background signal after His-tag staining (data not shown), we decided to use Nb32-44-hHH for the remaining experiments which can be detected via HA-tag. We investigated the localization of Nb32-44-hHH over a longer period and found that the bispecific nanobody remained on the cell surfaces up to 24 h. Yet, a slight reduction in signal intensity becomes apparent over time (**Figure 4A**). We used fluorescein-labeled dextran to stain the endolysosomal pathway and found no co-localization with Nb32-44-hHH (**Supplementary Figure 5**). The marginally reduced staining after 4 h and 24 h thus indicated dissociation of Nb32-44-hHH from ICAM-1 rather than internalization of the complexes. In the next step, cells were first incubated with Nb32-44-hHH and then with AF 488-labeled Bet v 1. After 30 min as well as after 4 h a clear co-localization of nanobodies (red) and Bet v 1 (green) on the cell surface can be observed as indicated by yellow coloring (**Figure 4B**). Additionally, after 4 h, isolated green spots were visible inside the cells, demonstrating partial internalization of Bet v 1 despite the presence of nanobodies. When cells were exposed to Bet v 1 without prior nanobody immobilization, we clearly noted a stronger internalization of the allergen (**Figure 4B**).

### The binding affinity of nanobody-antigen complexes is influenced by temperature

**Figure 5:**
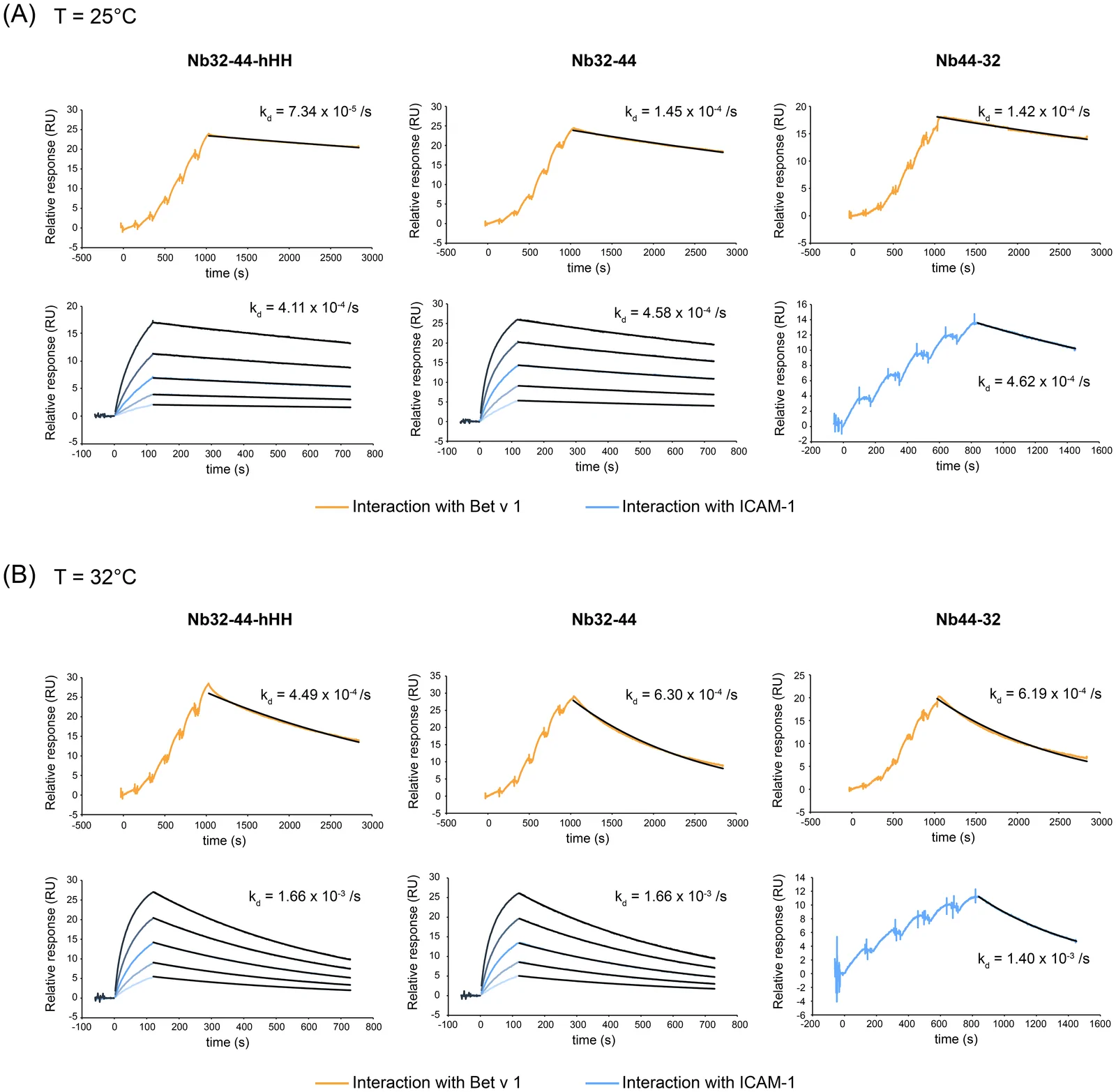
Binding kinetics of bispecific nanobodies to Bet v 1 and ICAM-1. The fitted dissociation rates (black) were superimposed onto the recorded data (in color) and calculated dissociation rate constants (k_d_) are indicated. Experiments were performed at **(A)** 25 °C and **(B)** 32 °C. Interactions between nanobodies and Bet v 1 (yellow) were investigated with the single-cycle kinetics mode employing Bet v 1 as immobilized ligand and nanobodies as analytes (between 1.5 nm and 948 nM). Interactions between nanobodies and ICAM-1 (blue) were investigated using Bet v 1-captured nanobodies as ligand and ICAM-1 as analyte (5 nm, 10 nm, 20 nm, 40 nm and 80 nM). All assays were performed twice in technical duplicates (except Nb44-32:ICAM-1, at 25 °C only once) and representative plots are shown.

To investigate a potential reason why Bet v 1 is not entirely retained at the cell surface by the Nb32-44hHH, we performed kinetic measurements. To gain knowledge about the half-life of the nanobody-antigen complexes, we run experiments at 25 °C, the commonly used standard measurement temperature for SPR analysis. We additionally included 32 °C to match the temperature used for the immunofluorescence staining. We confirmed simultaneous binding of both ICAM-1 and Bet v 1 to Nb32-44hHH and our other bispecific nanobody formats by observing increased signals after ICAM-1 injection over captured nanobodies on a Bet v 1 chip surface (**Figure 5**). At 25 °C, the dissociation rate constants (k_d_s) of Nb32-44-hHH, Nb32-44, Nb44–32 and Bet v 1 complexes (orange) ranged between 7.4 x 10^-5^/s and 1.5 x 10^-4^/s (**Figure 5A**, first row). The k_d_s of the interaction between the Bet v 1-captured nanobodies and the ICAM-1 (blue) were between 4.1 x 10^-4^/s and 4.6 x 10^-4^/s corresponding to a complex half-life of 2–4 h for Bet v 1 and 36–40 min for ICAM-1 (**Figure 5A**, second row). At 32 °C, we observed noteworthy faster k_d_s for both interactions, varying between 4.5 x 10^-4^/s and 6.3 x 10^-4^/s for Bet v 1:nanobody complexes (orange) and 1.4 x 10^-3^/s and 1.7 x 10^-3^/s for Bet v 1-bound nanobody:ICAM-1 complexes (blue), resulting in a half-life of around 30 min for Bet v 1 and 10–12 min for ICAM-1, respectively (**Figure 5B**). The raw recorded data are depicted in **Supplementary Figure 6**.

### Bispecific nanobodies reduce allergen penetration through an epithelial cell layer

**Figure 6:**
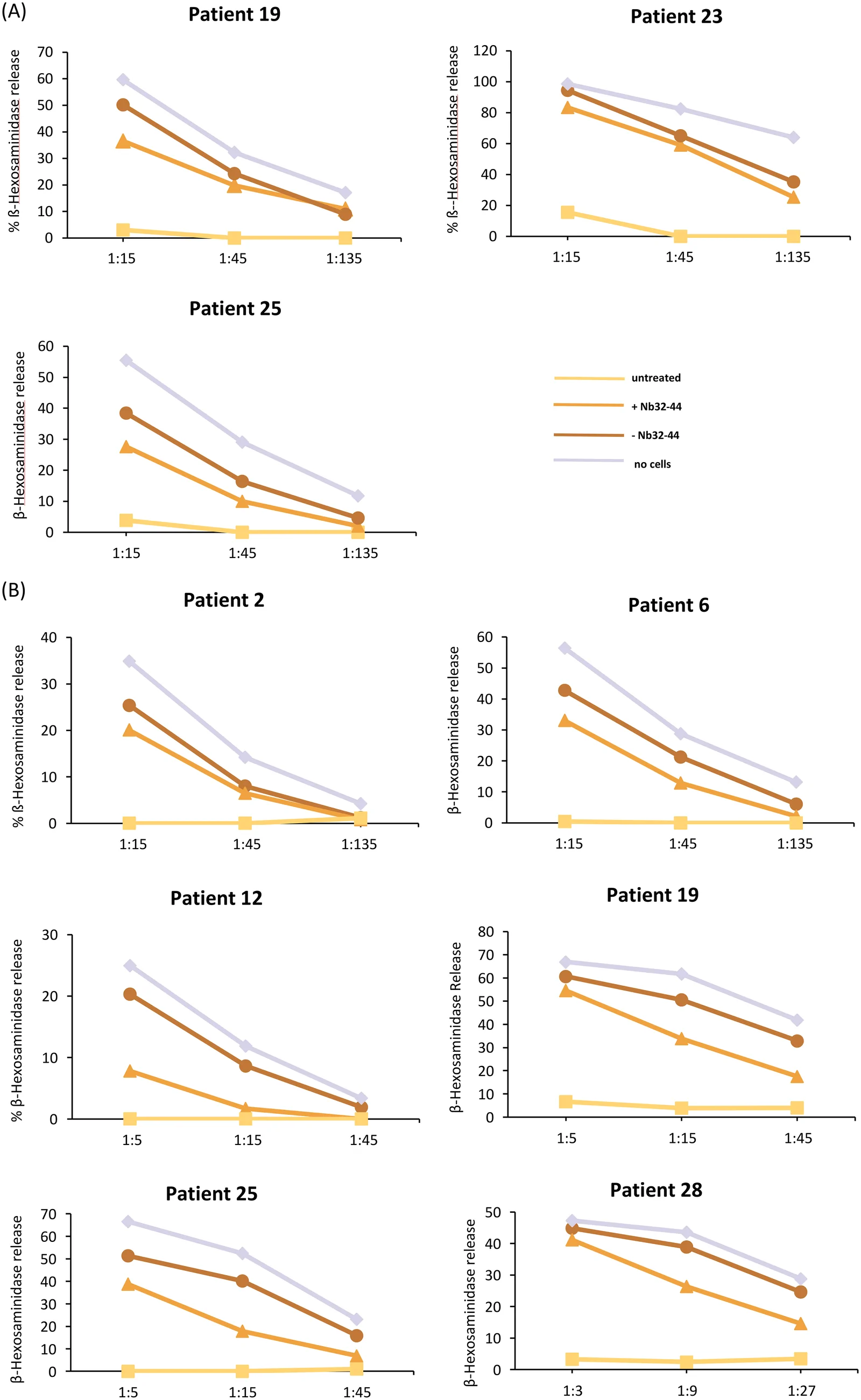
Degranulation of basophils induced by Bet v 1-containing basolateral media of transwell experiments. Rat basophilic leukemia (RBL) cells transfected with the human FcϵRI were loaded with IgE from birch pollen-allergic patients. Consequently, different dilutions (1:3 - 1:135) of basolateral medium from Bet v 1-exposed cell monolayers treated with **(A)** 500-fold or **(B)** 1000-fold excess (+) or without (–) Nb32-44-hHH, monolayers with intact tight junctions (untreated) or empty filters (no cells) were applied (x-axes). The percentage of Bet v 1-induced β-hexosaminidase release in relation to total β-hexosaminidase amount from lysed cells is displayed on the y-axes. Values are shown as means of technical duplicates of RBL assay. Displayed graphs are one representative of two independent transwell experiments.

To evaluate the potential of bispecific nanobodies to inhibit trans-epithelial allergen penetration, we performed *in vitro* transwell assays with 16HBE14o- cells and Nb32-44-hHH. Bispecific nanobodies were either applied or omitted from the EGTA-treated cells (i.e., cells with impaired barrier function) before exposure to Bet v 1. Cells without EGTA treatment (i.e., cells with intact barrier function) and transwells without cells (i.e., unhindered Bet v 1 flow) served as controls. The basolateral media were then applied to RBL cells loaded with IgE from several Bet v 1-sensitized patients and checked for their ability to induce degranulation. As expected, Bet v 1-induced degranulation was the highest when no cells were available in the apical compartment ranging from 25% to 100% (no cells, grey, dilution 1:5/1:15) (**Figure 6**). When the epithelial barrier remains intact, no ß-Hexosaminidase release was measured (untreated, yellow) (**Figure 6**). Noteably, basolateral media from impaired cell layers that had been pre-treated with a 500-fold excess of Nb32-44-hHH (**Figure 6A**) or a 1000-fold excess of Nb32-44-hHH (**Figure 6B**) elicited reduced release of ß-hexosaminidase (+Nb32-44-hHH, orange) compared to untreated impaired cells (-Nb32-44-hHH, brown). All tested patient sera showed this effect, although the extent varied markedly. β-Hexosaminidase release decreased by 10%–30% with a 500-fold excess of Nb32-44hHH (**Figure 6A**), whereas a 1000-fold excess reduced degranulation by up to 50% (**Figure 6B**).These results revealed that our bispecific nanobodies were able to diminish Bet v 1 penetration across the epithelial barrier but could not completely prevent it.

## Discussion

Prevention of allergic diseases represents a more effective public-health strategy than treating allergy. This proactive approach aims to stop clinically silent sensitization from progressing into IgE-mediated allergy and to minimize exposure to identified allergens. Since seasonal allergens occur only during a limited period each year, pollen allergy provides a well-defined natural exposure window. Importantly, administering interventions just prior to or at the very onset of this window targets the constitutive basal expression of epithelial adhesion molecules. The timely blockade prevents the early-stage mucosal priming and subsequent inflammatory cascade triggered by the initial wave of low-dose pollen exposure. This makes birch pollen allergy a suitable model to examine how reducing allergen contact within the respiratory tract and ocular mucosae influences allergic disease mechanisms.

In our study, we aimed to develop bispecific nanobodies for shielding respiratory epithelial cells to prevent allergen penetration through the epithelial barrier. This project represents a logical extension of our previous proof-of-concept study utilizing full-length IgG antibodies. Transitioning to nanobodies allows us to exploit their highly advantageous traits, such as easy production, lower manufacturing costs, high stability during nebulization and prolonged shelf life. These beneficial properties effectively compensate for the avidity advantages of conventional IgGs. However, if necessary the single-domain nature of nanobodies allows for the easy engineering of multi-valent or multi-epitopic constructs to gain higher avidity. To evaluate the feasibility of nanobodies for this purpose, we combined our previously characterized high-affine nanobodies specific for the major birch pollen allergen, Bet v 1 (Nb32) and the transmembrane protein, ICAM-1 (Nb44) (34, 35). Since ICAM-1 is strongly upregulated on the apical surface of epithelial cells under inflammatory conditions, we previously identified it as a suitable anchor for bispecific antibody conjugates (28, 29). The development of recombinant bispecific nanobodies represents a meaningful continuation of our initial efforts, as they are well-defined and highly controllable molecular tools. For our first bispecific nanobody formats we decided on head-to-tail combinations of Nb32 and Nb44 using the popular glycine-serine linker, allowing for flexible arrangement of the two monomers (42, 43). We designed three variations, designated Nb44-32, Nb32–44 and Nb32-44-hHH. By arranging the nanobodies both ways in respect to their N-terminal or C-terminal position, we found that one variant (Nb32-44) was better suited to bind to ICAM-1 on the cell surface. This finding verified the slightly increased ICAM-1-specific binding levels of Nb32–44 determined by ELISA. Our results support previous observations that both the order and the orientation (i.e., head-to-tail or tail-to-tail) of the antigen binding domains within a bispecific construct can significantly affect their affinity or biological activity towards their targets (44, 45). Another recent study provided evidence that longer linkers might yield better expression levels (46). While we kept the same linker length for all three constructs, we introduced the natural hinge of camel heavy chain antibodies between the C-terminus of the nanobody and the tags in one format (Nb32-44-hHH). This approach was in line with our earlier strategy (34, 35) and resulted in higher yields compared to its counterpart (Nb32-44).

When developing allergen-specific approaches for treating birch pollen allergy, it is important to consider cross-reactive allergens, since most Bet v 1-sensitized allergic patients additionally react to homologous allergens from the pathogenesis-related (PR) class 10 proteins like the major allergens from alder (Aln g 1) and hazel (Cor a 1) (1) For this reason, we highlighted both, the binding of our bispecific nanobodies to Bet v 1 and their ability to recognize Aln g 1 and Cor a 1. All three developed bispecific nanobodies bound to all three allergens, although Nb44–32 exhibited a slightly lower signal compared to Nb32–44 and Nb32-44-hHH. It remains unclear whether this results from an unfavorable arrangement of the DNA in the construct or from a weak expression quality of Nb44-32. However, when Nb44–32 was captured via immobilized Bet v 1, Aln g 1 or Cor a 1, ICAM-1 could be detected equally to captured Nb32–44 and Nb32-44-hHH. Due to these inconsistent results, we decided not to pursue a further characterization of Nb44–32 regarding cross-reactivity.

Since Nb44–32 also yielded inferior binding to surface-expressed ICAM-1 compared to Nb32-44, we only continued with Nb32–44 and Nb32-44-hHH. We confirmed that the immobilization of Bet v 1 to the surface is specifically achieved via the nanobodies. Although it has been shown that Bet v 1 can interact with membranes (47), we did not detect Bet v 1 signals when bispecific nanobodies were absent. We can therefore conclude that the Bet v 1 signal originated from binding to the nanobody rather than from unspecific attachment to the cell membrane. By initially performing the experiments at 4 °C – temperature that permits endocytic activity (48) – we also excluded the possibility of internalization of our molecules. Subsequent experiments performed at 32 °C to better reflect the physiological temperature of the nasal cavity revealed that topically applied Nb32-44-hHH remained bound to the cell surface. No internalization of Nb32-44-hHH was observed up to 24 h. This finding supports our earlier assumption that ICAM-1-specific nanobodies, including the bispecific format, do not cluster ICAM-1 on the cell surface and are therefore not endocytosed. When ICAM-1-immobilized Nb32-44-hHH was challenged to capture Bet v 1, we observed co-localization of the nanobodies and Bet v 1 at the cell membrane. In addition, Bet v 1 was also detectable in distinct intracellular spots after 4 h of exposure. This internalisation behavior aligns with recent insights into Bet v 1-epithelial interactions, providing the first evidence that Bet v 1 can actively penetrate healthy respiratory epithelial monolayers (49). Therefore, the observed intracellular localization after 4 hours likely reflects this inherent passive penetration kinetic, while the initial membrane co-localization highlights the capacity of our ICAM-1-targeted construct to successfully anchor and concentrate the incoming allergen at the luminal interface. Taken together, these results indicated that the bispecific nanobodies were able to keep Bet v 1 on the cell surface but did not fully prevent its uptake over an extended period. Faster dissociation rate constants of the Bet v 1:nanobody:ICAM-1 complexes at 32 °C compared with room temperature may account for this phenomenon. The increased temperature likely accelerates complex dissociation, reducing the ability to retain Bet v 1 at the cell surface. Nevertheless, we could show that Nb32-44-hHH was able to decrease Bet v 1 penetration. As a result, effector cell activation was reduced when compared to basolateral media from cell layers not loaded with Nb32-44-hHH. However, our present bispecific nanobody design still lags behind the performance of our previously generated bispecific antibody conjugates (28, 29). The fast dissociation rates of the protein complexes at 32 °C might be one explanation why Nb32-44-hHH is not able to fully prevent allergen penetration. To address this issue, we need to refine our current nanobody formats. We have therefore already implemented the following measures, which are currently being characterized and optimized. These approaches include a new immunization and selection process to generate 1.) nanobodies with slower dissociation rates from both antigens and 2.) nanobodies binding to non-overlapping epitopes on their specific antigens. In this context, it is worth mentioning new technologies allowing for computational design and selection of antigen binders. Synthetic nanobody libraries take the nanobody scaffold and introduce highly diverse complementarity-determining loops, thereby simulating the clonal diversity of an individual (50). Taking it one step further, a recent publication introduced a novel technology called BindCraft, which uses the protein structure model AlphaFold2 to predict, design, optimize and filter *de novo* binders (51). The authors developed a Bet v 1 binder that partially inhibited IgE binding from birch pollen allergic patients. This example successfully illustrates the development of a blocking, allergen-specific binder without relying on animal use or a high throughput screening process of the respective library (51). While computationally designed *de novo* binders offer exceptional precision, their *in vivo* translation necessitates a rigorous safety framework to address potential risk factors such as immunogenicity, off-target cross-reactivity driven by exposed hydrophobic patches, and rapid renal clearance.

For our next-generation bispecific nanobody platform, we envisage nanobodies with increased affinity or avidity. The latter may include tandem-linked nanobodies targeting different epitopes on the same antigen or post-translational multimerization of the current bispecific building block (46, 52). A bispecific multimer could also provide a certain degree of steric hindrance, which might have been an advantage of the successful bispecific antibody conjugates in the initial proof-of-concept studies (28, 29). Additionally, we initiated recloning of our nanobodies for mammalian expression with the aim of enhancing their stability and folding efficiency. Finally, a co-culture system to better represent of the respiratory epithelium might provide more accurate predictions on the functionality of the improved bispecific nanobody construct. It is known that besides paracellular penetration due to epithelial barrier damage (21, 53, 54), transcytosis is also an important factor in the uptake of Bet v 1 (15, 16, 55). Consequently, there are at least two modes of cellular entry that a topical treatment needs to inhibit. As topical delivery enable high local concentrations of protective nanobodies at the mucosal surface, it should be possible to prevent pollen allergen entry. We are confident that if we succeed in immobilizing bispecific nanobodies on the surface of the respiratory epithelium for several hours—preventing their rapid clearance by nasal secretions—one or two applications per day should be sufficient to maintain a stable nanobody shield. The key advantages of a local approach including rapid onset, reduced systemic exposure and hence reduced severe side effects, have already been acknowledged and led to development of first nanobodies for inhaled delivery tested in preclinical models (56, 57). Based on the knowledge gained from these pioneering studies, it should be feasible to advance the development of allergen-specific nanobodies for topical application and to test their efficacy in initial proof-of-concept studies.

When considering the clinical translation of topically applied bispecific constructs, potential interactions with non-epithelial targets and mucosal safety kinetics must be carefully evaluated. Although ICAM-1 is widely expressed on various resident immune cells, such as dendritic cells and macrophages, the epithelial layer remains the primary physical interface encountered by a daily administered nasal spray. Whether the protective nanobody shield targeting the compromised epithelial barrier of allergic patients efficiently intercepts incoming allergens at the luminal surface — thereby breaking the vicious cycle of barrier breakdown and subsequent immune cell activation — remains to be tested.

In addition to barrier integrity, the potential for local allergen accumulation on the mucosal surface warrants careful consideration. Recent data indicates that Bet v 1 can induce a transcriptional upregulation of alarmins like IL-33 in airway epithelium contributing to transcellular penetration (49). Therefore, for the successful design of an efficient apical nanobody shield on the respiratory epithelium, it is crucial to guarantee that captured Bet v 1 molecules do not merely accumulate at the cell surface and trigger downstream alarmin signaling. This molecular challenge can be strategically addressed via two distinct engineering pathways: first, by selecting an optimal epitope that directly blocks the specific allergen domains responsible for cell activation, and second, by optimizing the dissociation kinetics to regulate the clearance rate of the bound allergen complex from the luminal interface.

Beyond respiratory symptoms, Bet v 1 hypersensitivity is heavily linked to pollen-food allergy syndrome, most notably Mal d 1 from apples that triggers localized oral or systemic gastrointestinal allergic reactions. Given the high affinity of the bispecific nanobodies to Mal d 1 and related food allergens, the bispecific nanobody strategy presented here could theoretically be extended to dietary or oral supplements aimed at neutralizing cross-reactive food allergens. However, the therapeutic application of nanobodies within the gastrointestinal tract is strictly limited by the harsh proteolytic environment of the stomach and duodenum, where gastric acid and enzymes like pepsin and trypsin rapidly denature and digest unprotected proteins. To overcome this barrier and prevent allergen exposure through the gut mucosa, advanced pharmaceutical formulation strategies would be mandatory. These include enteric coatings, lipid nanoparticles, or microencapsulation to shield the nanobodies until they reach their target site. Alternatively, localized application to the oral mucosa via fast-dissolving lozenges or sublingual formulations could offer a viable route to neutralize cross-reactive food allergens directly in the oral cavity before digestion occurs.

With the current contribution, we succeeded for the first time to generate bispecific nanobodies that immobilize pollen allergens on the surface of epithelial cells and provided the first step to illustrate that bispecific nanobodies have potential for further development as topical agents for allergy treatment.

## Data Availability

The raw data supporting the conclusions of this article will be made available by the authors, without undue reservation.

## References

[1] Biedermann T Winther L Till SJ Panzner P Knulst A Valovirta E . Birch pollen allergy in Europe. Allergy. (2019) 74:1237–48. doi: 10.1111/all.13758 30829410

[2] Kwong KY Chen Z Scott L Hilborne LH . Optimizing identification of allergic sensitization to seasonal inhalant allergens in the USA: Implications for constructing optimal panels to evaluate patients with allergy. Int Arch Allergy Immunol. (2024) 185:848–55. doi: 10.1159/000538420 38781930

[3] Elisyutina O Lupinek C Fedenko E Litovkina A Smolnikov E Ilina N . IgE-reactivity profiles to allergen molecules in Russian children with and without symptoms of allergy revealed by micro-array analysis. Pediatr Allergy Immunol Off Publ Eur Soc Pediatr Allergy Immunol. (2021) 32:251–63. doi: 10.1111/pai.13354 32869350 PMC7891667

[4] Wang X Chen L Ding J Wang H Wang X . Profiles of birch allergen component sensitization and its association with pollen food allergy syndrome in Northern China. J Asthma Allergy. (2023) 16:1241–50. doi: 10.2147/JAA.S427764 38022747 PMC10656847

[5] Ellis AK DeVeaux M Steacy L Ramesh D Suprun M Langdon S . Environmental exposure unit simulates natural seasonal birch pollen exposures while maximizing change in allergic symptoms. Ann Allergy Asthma Immunol Off Publ Am Coll Allergy Asthma Immunol. (2021) 127:488–495.e5. doi: 10.1016/j.anai.2021.06.015 34186172

[6] Zuberbier T Lötvall J Simoens S Subramanian SV Church MK . Economic burden of inadequate management of allergic diseases in the European Union: a GA(2) LEN review. Allergy. (2014) 69:1275–9. doi: 10.1111/all.12470 24965386

[7] Kiotseridis H Cilio CM Bjermer L Aurivillius M Jacobsson H Dahl Å . Quality of life in children and adolescents with respiratory allergy, assessed with a generic and disease-specific instrument. Clin Respir J. (2013) 7:168–75. doi: 10.1111/j.1752-699X.2012.00298.x 22621438

[8] Jarolim E Rumpold H Endler AT Ebner H Breitenbach M Scheiner O . IgE and IgG antibodies of patients with allergy to birch pollen as tools to define the allergen profile of Betula verrucosa. Allergy. (1989) 44:385–95. doi: 10.1111/j.1398-9995.1989.tb04169.x 2802112

[9] Breiteneder H Kraft D . The history and science of the major birch pollen allergen Bet v 1. Biomolecules. (2023) 13:1151. doi: 10.3390/biom13071151 37509186 PMC10377203

[10] Raith M Swoboda I . Birch pollen-the unpleasant herald of spring. Front Allergy. (2023) 4:1181675. doi: 10.3389/falgy.2023.1181675 37255542 PMC10225653

[11] Kleine-Tebbe J Ballmer-Weber BK Breiteneder H Vieths S . Bet v 1 and its homologs: Triggers of tree-pollen allergy and birch pollen-associated cross-reactions. In: Kleine-Tebbe J Jakob T , editors. Molecular Allergy Diagnostics: Innovation for a Better Patient Management. Springer International Publishing, Cham (2017). p. 21–42.

[12] Mattila P Joenväärä S Renkonen J Toppila-Salmi S Renkonen R . Allergy as an epithelial barrier disease. Clin Transl Allergy. (2011) 1:5. doi: 10.1186/2045-7022-1-5 22410284 PMC3294629

[13] Leonardi A Motterle L Bortolotti M . Allergy and the eye. Clin Exp Immunol. (2008) 153:17–21. doi: 10.1111/j.1365-2249.2008.03716.x 18721324 PMC2515354

[14] Kortekaas Krohn I Seys SF Lund G Jonckheere AC Dierckx de Casterlé I Ceuppens JL . Nasal epithelial barrier dysfunction increases sensitization and mast cell degranulation in the absence of allergic inflammation. Allergy. (2020) 75:1155–64. doi: 10.1111/all.14132 31769882

[15] Joenväärä S Mattila P Renkonen J Mäkitie A Toppila-Salmi S Lehtonen M . Caveolar transport through nasal epithelium of birch pollen allergen Bet v 1 in allergic patients. J Allergy Clin Immunol. (2009) 124:135–142.e1-21. doi: 10.1016/j.jaci.2008.11.048 19344938

[16] Renkonen J Mattila P Lehti S Mäkinen J Sormunen R Tervo T . Birch pollen allergen Bet v 1 binds to and is transported through conjunctival epithelium in allergic patients. Allergy. (2009) 64:868–75. doi: 10.1111/j.1398-9995.2008.01919.x 19154545

[17] Nur Husna SM Tan HTT Md Shukri N Mohd Ashari NS Wong KK . Nasal epithelial barrier integrity and tight junctions disruption in allergic rhinitis: Overview and pathogenic insights. Front Immunol. (2021) 12:663626. doi: 10.3389/fimmu.2021.663626 34093555 PMC8176953

[18] Pothoven KL Schleimer RP . The barrier hypothesis and Oncostatin M: Restoration of epithelial barrier function as a novel therapeutic strategy for the treatment of type 2 inflammatory disease. Tissue Barriers. (2017) 5:e1341367. doi: 10.1080/21688370.2017.1341367 28665760 PMC5571776

[19] Sun N Ogulur I Mitamura Y Yazici D Pat Y Bu X . The epithelial barrier theory and its associated diseases. Allergy. (2024) 79:3192–237. doi: 10.1111/all.16318 39370939 PMC11657050

[20] Waltl EE Selb R Eckl-Dorna J Mueller CA Cabauatan CR Eiwegger T . Betamethasone prevents human rhinovirus- and cigarette smoke- induced loss of respiratory epithelial barrier function. Sci Rep. (2018) 8:9688. doi: 10.1038/s41598-018-27022-y 29946071 PMC6018698

[21] Van Cleemput J Poelaert KCK Laval K Impens F Van den Broeck W Gevaert K . Pollens destroy respiratory epithelial cell anchors and drive alphaherpesvirus infection. Sci Rep. (2019) 9:4787. doi: 10.1038/s41598-019-41305-y 30886217 PMC6423322

[22] Gill MA . The role of dendritic cells in asthma. J Allergy Clin Immunol. (2012) 129:889–901. doi: 10.1016/j.jaci.2012.02.028 22464668

[23] Lambrecht BN Hammad H . The role of dendritic and epithelial cells as master regulators of allergic airway inflammation. Lancet. (2010) 376:835–43. doi: 10.1016/S0140-6736(10)61226-3 20816550

[24] Royal C Gray C . Allergy prevention: An overview of current evidence. Yale J Biol Med. (2020) 93:689–98 33380931 PMC7757062

[25] Watts AM Cripps AW West NP Cox AJ . Modulation of allergic inflammation in the nasal mucosa of allergic rhinitis sufferers with topical pharmaceutical agents. Front Pharmacol. (2019) 10:294. doi: 10.3389/fphar.2019.00294 31001114 PMC6455085

[26] Bergmann KC Berger M Klimek L Pfaar O Werchan B Werchan M . Nonpharmacological measures to prevent allergic symptoms in pollen allergy: A critical review. Allergol Sel. (2021) 5:349–60. doi: 10.5414/ALX02294E 34870079 PMC8638355

[27] Bergmann KC Hartung T Zuberbier T . Individual wearable air purifier protects against pollen, house dust mite, and cat allergens: Report from an allergen exposure chamber. Allergol Sel. (2024) 8:70–7. doi: 10.5414/ALX02473E 38549812 PMC10975740

[28] Madritsch C Eckl-Dorna J Blatt K Ellinger I Kundi M Niederberger V . Antibody conjugates bispecific for intercellular adhesion molecule 1 and allergen prevent migration of allergens through respiratory epithelial cell layers. J Allergy Clin Immunol. (2015) 136:490–493.e11. doi: 10.1016/j.jaci.2015.01.006 25769914 PMC4530582

[29] Weichwald C Zettl I Ellinger I Niespodziana K Waltl EE Villazala-Merino S . Antibody conjugates bispecific for pollen allergens and ICAM-1 with potential to prevent epithelial allergen transmigration and rhinovirus infection. Int J Mol Sci. (2023) 24:2725. doi: 10.3390/ijms24032725 36769047 PMC9917280

[30] Ciprandi G Buscaglia S Pesce G Villaggio B Bagnasco M Canonica GW . Allergic subjects express intercellular adhesion molecule--1 (ICAM-1 or CD54) on epithelial cells of conjunctiva after allergen challenge. J Allergy Clin Immunol. (1993) 91:783–92. doi: 10.1016/0091-6749(93)90198-o 8095941

[31] Ciprandi G Pronzato C Ricca V Bagnasco M Canonica GW . Evidence of intercellular adhesion molecule-1 expression on nasal epithelial cells in acute rhinoconjunctivitis caused by pollen exposure. J Allergy Clin Immunol. (1994) 94:738–46. doi: 10.1016/0091-6749(94)90182-1 7930308

[32] Muyldermans S . A guide to: Generation and design of nanobodies. FEBS J. (2021) 288:2084–102. doi: 10.1111/febs.15515 32780549 PMC8048825

[33] Alexander E Leong KW . Discovery of nanobodies: A comprehensive review of their applications and potential over the past five years. J Nanobiotechnology. (2024) 22:661. doi: 10.1186/s12951-024-02900-y 39455963 PMC11515141

[34] Zettl I Ivanova T Strobl MR Weichwald C Goryainova O Khan E . Isolation of nanobodies with potential to reduce patients’ IgE binding to Bet v 1. Allergy. (2022) 77:1751–60. doi: 10.1111/all.15191 34837242

[35] Zettl I Ivanova T Zghaebi M Rutovskaya MV Ellinger I Goryainova O . Generation of high affinity ICAM-1-specific nanobodies and evaluation of their suitability for allergy treatment. Front Immunol. (2022) 13:1022418. doi: 10.3389/fimmu.2022.1022418 36439110 PMC9682242

[36] Nakamura R Uchida Y Higuchi M Nakamura R Tsuge I Urisu A . A convenient and sensitive allergy test: IgE crosslinking-induced luciferase expression in cultured mast cells. Allergy. (2010) 65:1266–73. doi: 10.1111/j.1398-9995.2010.02363.x 20374229 PMC3066406

[37] Bauernfeind C Zettl I Ivanova T Goryainova O Weijler AM Pranz B . Trimeric Bet v 1-specific nanobodies cause strong suppression of IgE binding. Front Immunol. (2024) 15:1343024. doi: 10.3389/fimmu.2024.1343024 38784378 PMC11112410

[38] Kozak M . Comparison of initiation of protein synthesis in procaryotes, eucaryotes, and organelles. Microbiol Rev. (1983) 47:1–45. doi: 10.1128/mr.47.1.1-45.1983 6343825 PMC281560

[39] Keck T Leiacker R Riechelmann H Rettinger G . Temperature profile in the nasal cavity. Laryngoscope. (2000) 110:651–4. doi: 10.1097/00005537-200004000-00021 10764013

[40] Palladino C Ellinger I Kalic T Humeniuk P Ret D Mayr V . Peanut lipids influence the response of bronchial epithelial cells to the peanut allergens Ara h 1 and Ara h 2 by decreasing barrier permeability. Front Mol Biosci. (2023) 10:1126008. doi: 10.3389/fmolb.2023.1126008 36845549 PMC9945344

[41] Panou DA Pedersen SF Kristensen M Nielsen HM . Epithelium dynamics differ in time and space when exposed to the permeation enhancers penetramax and EGTA. A head-to-head mechanistic comparison. Front Drug Delivery. (2023) 3:1221628. doi: 10.3389/fddev.2023.1221628 40838052 PMC12363289

[42] De Vlieger D Ballegeer M Rossey I Schepens B Saelens X . Single-domain antibodies and their formatting to combat viral infections. Antibodies. (2018) 8:1. doi: 10.3390/antib8010001 31544807 PMC6640686

[43] Bannas P Hambach J Koch-Nolte F . Nanobodies and nanobody-based human heavy chain antibodies as antitumor therapeutics. Front Immunol. (2017) 8:1603. doi: 10.3389/fimmu.2017.01603 29213270 PMC5702627

[44] Asano R Kumagai T Nagai K Taki S Shimomura I Arai K . Domain order of a bispecific diabody dramatically enhances its antitumor activity beyond structural format conversion: The case of the hEx3 diabody. Protein Eng Des Sel PEDS. (2013) 26:359–67. doi: 10.1093/protein/gzt009 23468569

[45] Hultberg A Temperton NJ Rosseels V Koenders M Gonzalez-Pajuelo M Schepens B . Llama-derived single domain antibodies to build multivalent, superpotent and broadened neutralizing anti-viral molecules. PloS One. (2011) 6:e17665. doi: 10.1371/journal.pone.0017665 21483777 PMC3069976

[46] Tillib SV Goryainova OS . Extending linker sequences between antigen-recognition modules provides more effective production of bispecific nanoantibodies in the periplasma of E. coli. Biochem Biokhimiia. (2024) 89:933–41. doi: 10.1134/S0006297924050134 38880653

[47] Mogensen JE Ferreras M Wimmer R Petersen SV Enghild JJ Otzen DE . The major allergen from birch tree pollen, Bet v 1, binds and permeabilizes membranes. Biochemistry. (2007) 46:3356–65. doi: 10.1021/bi062058h 17311414

[48] Silverstein SC Steinman RM Cohn ZA . Endocytosis. Annu Rev Biochem. (1977) 46:669–722. doi: 10.1146/annurev.bi.46.070177.003321 332066

[49] Melnikova DN Potapov AE Ovchinnikova TV Bogdanov IV . Modeling human airway epithelial barrier penetration using birch Bet v 1 and alder Aln g 1 pollen allergens during sensitization process. Int J Mol Sci. (2025) 26:5169. doi: 10.3390/ijms26115169 40507979 PMC12155471

[50] Contreras MA Serrano-Rivero Y González-Pose A Salazar-Uribe J Rubio-Carrasquilla M Soares-Alves M . Design and construction of a synthetic nanobody library: Testing its potential with a single selection round strategy. Molecules. (2023) 28:3708. doi: 10.3390/molecules28093708 37175117 PMC10180287

[51] Pacesa M Nickel L Schellhaas C Schmidt J Pyatova E Kissling L . One-shot design of functional protein binders with BindCraft. Nature. (2025) 646:483–92. doi: 10.1038/s41586-025-09429-6 40866699 PMC12507698

[52] Deiteren A Krupka E Bontinck L Imberdis K Conickx G Bas S . A proof-of-mechanism trial in asthma with lunsekimig, a bispecific NANOBODY molecule. Eur Respir J. (2025) 65:2401461. doi: 10.1183/13993003.01461-2024 39884759 PMC12018761

[53] Gangl K Waltl EE Vetr H Cabauatan CR Niespodziana K Valenta R . Infection with rhinovirus facilitates allergen penetration across a respiratory epithelial cell layer. Int Arch Allergy Immunol. (2015) 166:291–6. doi: 10.1159/000430441 26044772 PMC6536380

[54] Yazici D Ogulur I Pat Y Babayev H Barletta E Ardicli S . The epithelial barrier: The gateway to allergic, autoimmune, and metabolic diseases and chronic neuropsychiatric conditions. Semin Immunol. (2023) 70:101846. doi: 10.1016/j.smim.2023.101846 37801907

[55] Akgün J Schabussova I Schwarzer M Kozakova H Kundi M Wiedermann U . The role of alveolar epithelial type II-like cells in uptake of structurally different antigens and in polarisation of local immune responses. PloS One. (2015) 10:e0124777. doi: 10.1371/journal.pone.0124777 25894334 PMC4404363

[56] Gai J Ma L Li G Zhu M Qiao P Li X . A potent neutralizing nanobody against SARS-CoV-2 with inhaled delivery potential. MedComm. (2021) 2:101–13. doi: 10.1002/mco2.60 33821254 PMC8013425

[57] Zhu M Ma L Gai J Li G Huang J Li X . Preclinical and phase I randomized study of a potent inhalable nanobody targeting TSLP for the treatment of airway inflammation. J Allergy Clin Immunol. (2025) 156:1314–1327.e7. doi: 10.1016/j.jaci.2025.06.024 40602691

