# Correction: Bispecific nanobodies targeting Bet v 1 and ICAM-1 decrease respiratory epithelial penetration of Bet v 1

**DOI:** 10.3389/fimmu.2026.1974800

**Published:** 2026-09-11

**Authors:** Ines Zettl, Isabella Ellinger, Mohammed Zghaebi, Sarah Jance, Vanessa Izewski, Anja Drescher, Heimo Breiteneder, Manuel Röck, Martin Tollinger, Julia Eckl-Dorna, Sergei V. Tillib, Sabine Flicker

**Affiliations:** 1 Institute of Pathophysiology and Allergy Research, Center for Pathophysiology, Infectiology and Immunology, Medical University of Vienna, Vienna, Austria; 2 Vienna Airway Lab, Department of Otolaryngology, Head and Neck Surgery, Medical University of Vienna, Vienna, Austria; 3 Cytiva Europe GmbH, Freiburg, Germany; 4 Department of Organic Chemistry, University of Innsbruck, Innsbruck, Austria; 5 Institute of Gene Biology, Russian Academy of Sciences, Moscow, Russia

**Keywords:** birch pollen allergy, bispecific nanobody, ICAM-1, respiratory epithelium, topical treatment

Reference 49 “Melnikova DN, Potapov AE, Ovchinnikova TV, Bogdanov IV. Modeling human airway epithelial barrier penetration using birch Bet v 1 and alder Aln g 1 pollen allergens during sensitization process. *Int J Mol Sci.* (2025) 26:5169. doi: 10.3390/ijms26115169” was not cited in the article. The citation has now been inserted in the section **Discussion**, paragraph 4 and should read:

“This internalisation behavior aligns with recent insights into Bet v 1-epithelial interactions, providing the first evidence that Bet v 1 can actively penetrate healthy respiratory cell epithelial monolayers (49).”

Consequently, the sequence of all subsequent references has been changed as shown below:

“Synthetic nanobody libraries take the nanobody scaffold and introduce highly diverse com plementarity-determining loops, thereby simulating the clonal diversity of an individual (50). Taking it one step further, a recent publication introduced a novel technology called BindCraft, which uses the protein structure model AlphaFold2 to predict, design, optimize and filter *de novo* binders (51).”

“This example successfully illustrates the development of a blocking, allergen-specific binder without relying on animal use or a high throughput screening process of the respective library (51).”

“The latter may include tandem-linked nanobodies targeting different epitopes on the same antigen or post-translational multimerization of the current bispecific building block (46, 52).”

“It is known that besides paracellular penetration due to epithelial barrier damage (21, 53, 54), transcytosis is also an important factor in the uptake of Bet v 1 (15, 16, 55).”

“The key advantages of a local approach including rapid onset, reduced systemic exposure and hence reduced severe side effects, have already been acknowledged and led to development of first nanobodies for inhaled delivery tested in preclinical models (56, 57).”

“Recent data indicates that Bet v 1 can induce a transcriptional of alarmins like IL-33 in airway epithelium contributing to transcellular penetration (49).”

