# Supplementary material for: Bispecific nanobodies targeting Bet v 1 and ICAM-1 decrease respiratory epithelial penetration of Bet v 1
Source: Front Immunol. 2026 Aug 17;17:1864741. doi: 10.3389/fimmu.2026.1864741 (PMC13527034; doi:10.3389/fimmu.2026.1864741)
Supplement: Supplementary file 4 [file Image4.pdf]

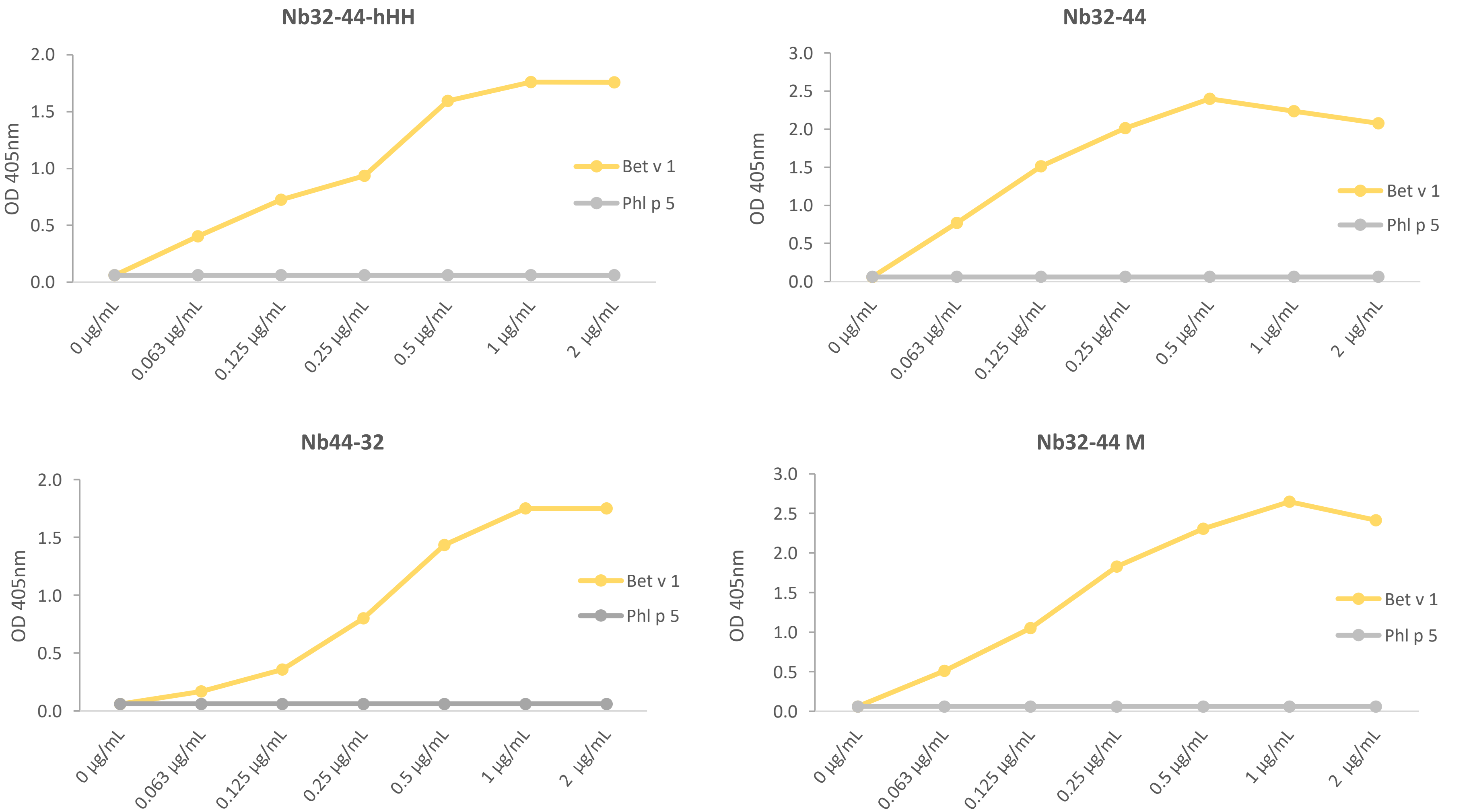

Zettl et al. Supplemental Figure 4A

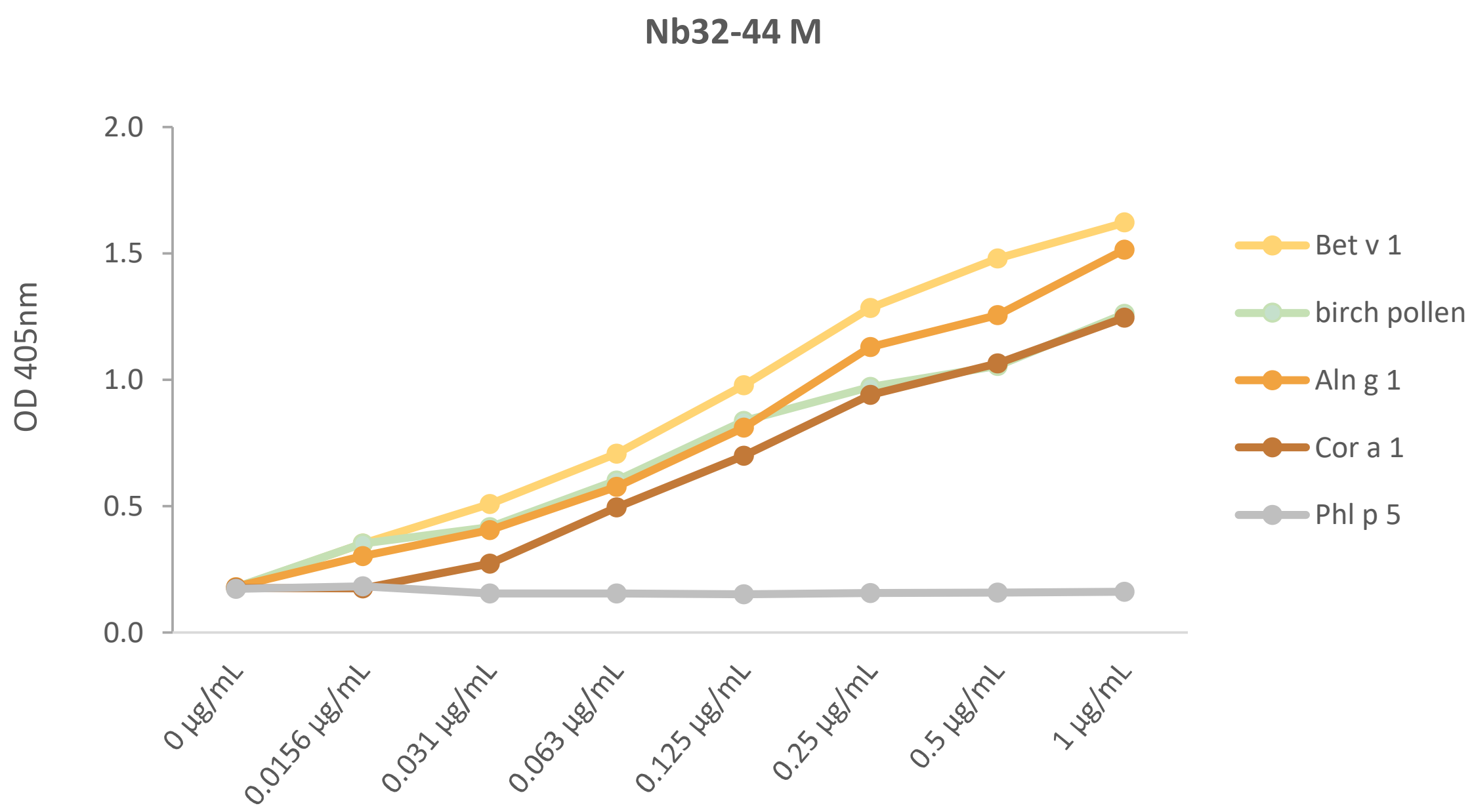

Zettl et al. Supplemental Figure 4B
