# Supplementary material for: Bispecific nanobodies targeting Bet v 1 and ICAM-1 decrease respiratory epithelial penetration of Bet v 1
Source: Front Immunol. 2026 Aug 17;17:1864741. doi: 10.3389/fimmu.2026.1864741 (PMC13527034; doi:10.3389/fimmu.2026.1864741)
Supplement: Supplementary file 7 [file DataSheet1.docx]

**Bispecific nanobodies targeting Bet v 1 and ICAM-1 decrease respiratory epithelial penetration of Bet v 1**

Ines Zettl^1^, Isabella Ellinger^1^, Mohammed Zghaebi^2^, Sarah Jance^1^, Vanessa Izewski^1^, Anja Drescher^3^, Heimo Breiteneder^1^, Manuel Röck^4^, Martin Tollinger^4^, Julia Eckl-Dorna^2^, Sergei V. Tillib^4*^, Sabine Flicker^1*‡^

^1^Institute for Pathophysiology and Allergy Research, Center for Pathophysiology, Infectiology and Immunology, Medical University of Vienna, Vienna, Austria

**^2^Vienna Airway Lab, Department of Otolaryngology, Head and Neck Surgery, Medical University of Vienna, Vienna, Austria**

^3^Cytiva Europe GmbH, Freiburg, Germany

^4^Department of Organic Chemistry, University of Innsbruck, Innsbruck, Austria

^5^Institute of Gene Biology, Russian Academy of Sciences, Moscow, Russia

*Co-last authors: Sergei V. Tillib and Sabine Flicker

^‡^Corresponding author: Sabine Flicker, Institute for Pathophysiology and Allergy Research, Center for Pathophysiology, Infectiology and Immunology, Medical University of Vienna, Vienna, Austria; Phone: +43 1-40400-51260; Fax: +43 1-40400-51300; Email: sabine.flicker@meduniwien.ac.at

**MATERIAL and METHODS**

**Nb32-44 M design, expression and purification**

DNA sequences of Nb32 (GenBank accession number MZ708607) and Nb44 (GenBank accession number ON950079) were codon-optimized for mammalian expression and were synthesized by Twist Bioscience (San Francisco, CA, USA). They were linked head-to-tail with a standard (GGGGS)_3_ linker and inserted into the pCSE2.5 expression vector upstream of the HA, Avi and 6x His-Tag and expressed in HEK-6E cells. Purification was by HIS-Select Nickel affinity chromatography (Sigma-Aldrich).

**SDS-PAGE**

Sodium dodecyl sulfate polyacrylamide gel electrophoresis (SDS-PAGE) was performed as described in the manuscript. Purified Nb32-44 M (2 µg/slot) was separated on a 14% polyacrylamide gel under reducing and non-reducing conditions. Protein bands were stained with InstantBlue Coomassie Protein Stain (Abcam, Cambridge, UK). The PageRuler™ Plus Prestained Protein Ladder, 10 to 250 kDa from Thermo Fisher Scientific (Cat. 26619) was used for size comparison.

**Reactivity of Nb32-44 M (ELISA)**

Allergens (Bet v 1, Aln g 1, Cor a 1 and Phl p 5; 2 µg/mL) and birch pollen extract (10 µg/mL) were coated onto ELISA plates (MaxiSorp, Nunc, Roskilde, Denmark) diluted in 0.1 M bicarbonate buffer overnight at 4°C. After washing with PBS containing 0.05% Tween20 (PBST), plates were blocked with 1% BSA in PBST and afterwards incubated with two-fold serial dilutions of Nb32-44 M (ranging from 0.0156 µg/mL to 1 µg/mL) in 0.1% BSA in PBST overnight at 4°C. Bound nanobodies were detected with HRP-labeled mouse anti-His-tag antibodies (Cat. MA1-21315, Invitrogen) diluted 1:4,000 in 0.1% BSA in PBST. After repeated washing, HRP substrate 2,2'-azino-bis(3-ethylbenzothiazoline-6-sulfonic acid) (ABTS) (Cat. A1888, Sigma-Aldrich) was added to achieve the color reaction. ODs at 405 nm (reference wavelength: 495 nm) were measured with a TECAN Infinite F50 microplate reader (Männedorf, Switzerland).

**RESULTS**

**Nb32-44 M expression**

The average expression yield of Nb32-44 M was 4 mg/150 mL. The calculated molecular mass and theoretical isoelectrical point based on the amino acid sequence are 32.1 kDa and 6.05. Analysis of purified Nb32-44 M by reducing and non-reducing SDS-PAGE revealed one band at expected size of 32 kDa (Supplemental Figure 2D). No signs of aggregation or degradation were observed.

**Nb32-44 M binds to Bet v 1 and Bet v 1 homologs**

In addition to recombinant Bet v 1, Nb32-44 M recognized natural Bet v 1 within birch pollen extract, as well as Aln g 1 and Cor a 1. It exhibited no binding to Phl p 5, thereby demonstrating the high specificity of this construct (Supplemental Figure 4 A, B)

**Table S1:** Antibodies, endocytic marker and fluorescent dyes used in Western blot (WB), ELISA, flow cytometry (FC) and immunofluorescence microscopy (IFM). HRP, horseradish peroxidase, PE, phycoerythrin

| **Antibodies, endocytic marker or fluorescent dyes** | **Conjugate** | **Company** | **Clone** | **Cat. no** | **used for** |
| --- | --- | --- | --- | --- | --- |
| Anti-His-tag antibody | HRP | Invitrogen | polyclonal | PA1-23024 | WB |
| Anti-His-tag antibody | HRP | Invitrogen | monoclonal | **MA1-21315** | ELISA |
| Anti-His-tag antibody | PE | Biolegend | J095G46 | 362603 | FC |
| Anti-HA-tag antibody | unconjugated | Invitrogen | 2-2.2.14 | 26183 | IFM |
| Anti-mouse antibody | Alexa Fluor 568 | Invitrogen | polyclonal | A-11004 | IFM |
| Anti-rabbit antibody | HRP | Sigma | polyclonal | SAB3700934 | ELISA |
| Fixable viability dye eFluor 780 | unconjugated | ThermoFisher Scientific |  | 65-0865-14 | FC |
| Dextran 40 kDa, lysine-fixable | Fluorescein | ThermoFisher Scientific |  | D1845 | IFM |
| DRAQ5 | unconjugated | ThermoFisher Scientific |  | 62254 | IFM |

**Table S 2:** Sequence alignment of amino acid sequences from Bet v 1 (Uniprot: P15494) and cross-reactive pollen allergens Aln g 1 (Uniprot: P38948) and Cor a 1 (Uniprot: Q08407). Points indicate identical amino acids and sequence identities are displayed as percentages below.

```
Bet v 1         MGVFNYETETTSVIPAARLFKAFILDGDNLFPKVAPQAISSVENIEGNGGPGTIKKISFP
Aln g 1         **********P*************************E*V******************T**
Cor a 1         *******V**P**********SYV****K*I********T****V**********N*T*G


Bet v 1         EGFPFKYVKDRVDEVDHTNFKYNYSVIEGGPIGDTLEKISNEIKIVATPDGGSILKISNK
Aln g 1         **S******E******RV****SF******AV**A***VC*******A************
Cor a 1         **SRY****E******N***T*S*T****DVL**K***VCH*L****A*G********S*


Bet v 1           YHTKGDHEVKAEQVKASKEMGETLLRAVESYLLAHSDAYN
Aln g 1           F*******IN***I*IE**KAVG**K**************
Cor a 1           F*A*****IN**EM*GA***A*K******T******AE**
```

**Sequence Identity**

Bet v 1 – Aln g 1: 133 AA = 83%

Bet v 1 – Cor a 1: 116 AA = 73%

**Supplemental Figure 1.** Sequential gating strategy for the flow cytometric detection of His-tagged nanobodies immobilized on the surface of 16HBE14o- cells (A) and of surface-bound nanobodies, capturing Bet v 1 on those cells (B). Plots are arranged left to right in the order of gating: events were first gated on FSC-A versus SSC-A to exclude debris, then on single cells, and then on live cells. Nanobodies were detected with an anti-His tag–PE conjugate (y-axes in A and B) and are displayed against AF 647-labelled Bet v 1 (x-axis, B).

**Supplemental Figure 2.** Coomassie-Blue stained SDS-PAGE analysis of the Nb32-44 (A), Nb44-32 (B) and Nb32-44-hHH (C) purification process under reducing conditions. M, molecular weights in kDa; SN, supernatant; PE, periplasmatic extract; FT, flow-through; and E1-7, elution fractions are indicated. (D) Coomassie-Blue stained SDS-PAGE analysis of purified Nb32-44 M under reducing (+) and non-reducing (-) conditions.

**Supplemental Figure 3.** Binding capacity of bispecific nanobodies Nb32-44-hHH, Nb32-44 and Nb44-32 as determined by ELISA. (A) Reactivity of ICAM-1 (blue) with bispecific nanobodies that were pre-bound to coated allergens (displayed on the x-axes). When the second antigen (i.e., ICAM-1) or the bispecific nanobodies were omitted, no signal could be detected (grey). (B) Reactivity of Bet v 1 (yellow), Aln g 1 (orange) or Cor a 1 (brown) with bispecific nanobodies that were pre-bound to coated ICAM-1. When the second antigen (i.e., Bet v 1, Aln g 1 or Cor a 1) or the bispecific nanobodies were omitted, no signal could be detected (grey). OD-values correspond to the amount of bound second antigens and are depicted as means of technical triplicates ± standard deviation (SD).

**Supplemental Figure 4.** (A) Binding capacity of two-fold serial dilutions of bispecific Nb32-44-hHH, Nb32-44, Nb44-32 and Nb32-44 M to Bet v 1 and the unrelated major grass pollen allergen, Phl p 5. (B) Binding capacity of two-fold serial dilutions of bispecific Nb32-44 M to Bet v 1, birch pollen extract, Aln g 1, Cor a 1 and the unrelated major grass pollen allergen, Phl p 5. OD-values correspond to the amount of bound nanobodies and are depicted as means of technical duplicates.

**Supplemental Figure 5.** Confocal immunofluorescence microscopy of 16HBE14o- cells showing localization of Nb32-44-hHH, Nb32 and Nb44 (red) and the endolysosomal marker dextran (green). Nb32-44-hHH and Nb44 remained bound to the cell surface while dextran was internalized. 16HBE14o- cell were simultaneously incubated with nanobodies and fluorescein-labeled dextran at 32 °C for the indicated duration. For 4h and 24h, dextran was added during the last 30 min of the experiments. After cell fixation, nanobodies were detected with mouse anti-HA-tag antibodies and stained with AF568-labeled anti-mouse antibodies. The white scale bar in the bottom left corners corresponds to 10 µm.

**Supplemental Figure 6.** Raw data plots of kinetic measurements depicted in Figure 5. Experiments were performed at **(A)** 25 °C or **(B)** 32 °C. Interaction with Bet v 1 (orange and red tones) was investigated with the single cycle kinetic mode. Curves are depicted in reference to a buffer cycle (black). Nb32 served as positive control, Nb44 served as negative control. Bispecific nanobodies were injected in three-fold dilutions (ranging from 1.5 nM to 948 nM) as technical duplicates. Assays were performed two times (Bet v 1:nanobody) in technical duplicates and representative plots are shown.

Interaction with ICAM-1 (blue tones) was investigated with the multi-cycle (Nb32-44-hHH, Nb32-44) or single-cycle (Nb44-32) kinetic mode. Nanobodies (250 nM, 186 nM and 316 nM, respectively) were captured on the Bet v 1-chip surface to a level of 15 – 30 RU (first injection peak). Afterwards, ICAM-1 (5 nM – 80 nM, two-fold dilutions) was injected either after each nanobody injection (multi-cycle) or sequentially in one measurement (single-cycle). Buffer was injected over the captured nanobody surface for referencing the ICAM-1 injections (light grey). For control purposes, buffer or ICAM-1 (80 nM) were injected on the Bet v 1 surface without nanobodies (purple curves). All assays were performed twice in technical duplicates (except Nb44-32:ICAM-1, at 25 °C only once) and representative plots are shown.
